# Cadherin and Wnt signaling pathways as key regulators in diabetic nephropathy

**DOI:** 10.1371/journal.pone.0255728

**Published:** 2021-08-19

**Authors:** Maria Tziastoudi, Aspasia Tsezou, Ioannis Stefanidis

**Affiliations:** 1 Department of Nephrology, School of Medicine, University of Thessaly, Larissa, Greece; 2 Laboratory of Biology, Faculty of Medicine, School of Health Sciences, University of Thessaly, Larissa, Greece; 3 Laboratory of Cytogenetics and Molecular Genetics, Faculty of Medicine, School of Health Sciences, University of Thessaly, Larissa, Greece; University of Southern California, UNITED STATES

## Abstract

**Aim:**

A recent meta-analysis of genome-wide linkage studies (GWLS) has identified multiple genetic regions suggestive of linkage with DN harboring hundreds of genes. Moving this number of genetic loci forward into biological insight is truly the next step. Here, we approach this challenge with a gene ontology (GO) analysis in order to yield biological and functional role to the genes, an over-representation test to find which GO terms are enriched in the gene list, pathway analysis, as well as protein network analysis.

**Method:**

GO analysis was performed using protein analysis through evolutionary relationships (PANTHER) version 14.0 software and P-values less than 0.05 were considered statistically significant. GO analysis was followed by over-representation test for the identification of enriched terms. Statistical significance was calculated by Fisher’s exact test and adjusted using the false discovery rate (FDR) for correction of multiple tests. Cytoscape with the relevant plugins was used for the construction of the protein network and clustering analysis.

**Results:**

The GO analysis assign multiple GO terms to the genes regarding the molecular function, the biological process and the cellular component, protein class and pathway analysis. The findings of the over-representation test highlight the contribution of cell adhesion regarding the biological process, integral components of plasma membrane regarding the cellular component, chemokines and cytokines with regard to protein class, while the pathway analysis emphasizes the contribution of Wnt and cadherin signaling pathways.

**Conclusions:**

Our results suggest that a core feature of the pathogenesis of DN may be a disturbance in Wnt and cadherin signaling pathways, whereas the contribution of chemokines and cytokines need to be studied in additional studies.

## Introduction

Diabetic nephropathy (DN) is a multifactorial disease caused by both genetic and environmental factors [1, 2]. The functional and structural kidney injury in patients with diabetes is the result of alterations in both hemodynamic and metabolic factors, as well as inflammatory molecules and pathways [3–5]. The genetic background of DN has not been elucidated precisely yet, although multiple genetic factors have been implicated in the pathogenesis of the disease [5–8]. A recent meta-analysis of genetic association studies regarding 606 variants located in 228 genes highlighted the contribution of 66 genetic variants harbored in 53 genes [9].

Another type of studies for the genetic dissection of complex traits is the conduct of genome-wide linkage studies (GWLS) [10, 11]. Linkage studies of complex traits frequently yield a relatively large number of genetic regions suggestive of linkage harboring an impressive number of genes. One of the challenges in the analysis of large gene lists is unraveling the biological and functional role of these genes [12, 13]. GWLS in DN, as well as meta-analyses if these studies, have also identified numerous genetic regions suggestive of linkage with DN, although the results are inconclusive [14, 15]. Moving forward this impressive number of genetic loci into the underlying biology is the challenge. One way for the identification and prioritization of the most relevant cellular processes and pathways affected by the multiple genes is the gene ontology (GO) analysis [16, 17].

The Gene Ontology resource (GO; http://geneontology.org) provides structured, computable knowledge regarding the functions of genes and gene products [16, 17]. The ontology covers three distinct aspects of gene function: molecular function (the biochemical activity including specific binding to ligands or structures of a gene product), biological process (a biological objective to which the gene or gene product contributes) and cellular component (the place in the cell where a gene product is active) [16, 17].

In effort to analyze the results of the most recent meta-analysis of GWLS in DN [15] and reveal the underlying biology of the genetic loci located in statistical significant genetic regions, we performed a gene ontology analysis followed by an over-representation test for the identification of enriched GO terms, we constructed the protein network analysis for the identifications of hub genes and finally, the prioritization of candidate genes for further study, a similar approach of Shriner et al. [18].

## Materials and methods

### Data sources

**Fig 1:**
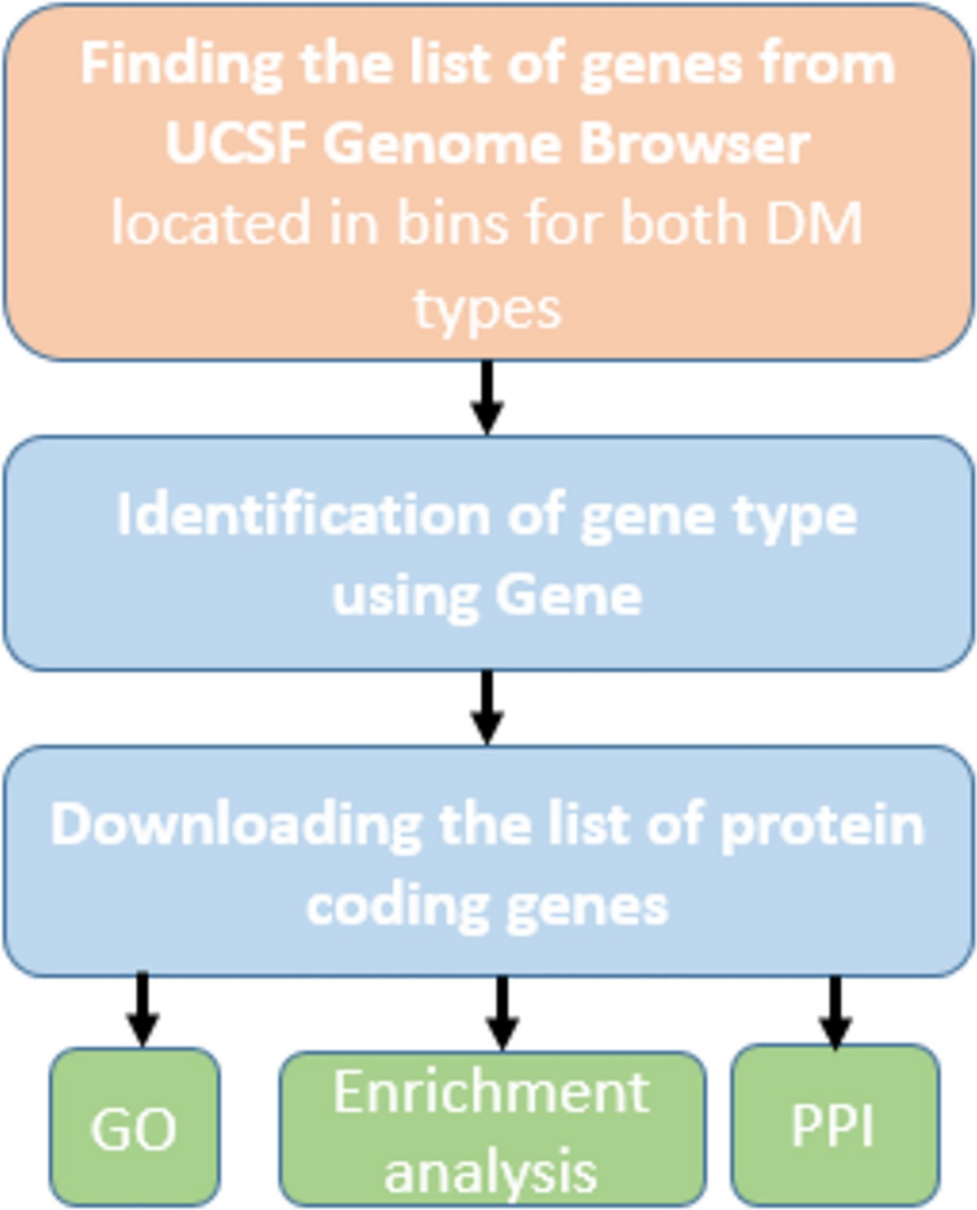
Study design flowchart.

In the present study, the data were derived from a meta-analysis of GWLS in DN [15]. DN was defined on the basis of a long-standing diabetes mellitus, either T1D or T2D, with macroalbuminuria and/or chronic renal insufficiency in the absence of nondiabetic renal disease. GWLS with quantitative surrogate markers for DN such as estimated glomerular filtration rate (eGFR), albuminuria and serum creatinine were excluded from the meta-analysis. In the meta-analysis probands with DN from 1833 families were included [15]. We identified the genes located in the statistical significant cytogenetic regions from meta-analysis using the University of California Santa Cruz (UCSC) Genome Browser (https://genome.ucsc.edu/) and more particularly, the assembly Dec. 2013 (GRCh38/hg38) [19]. Main meta-analysis identified seven genetic regions (4p14–4q13.3, 5q14.3–5q23.2, 5q23.2–5q34, 15p13–15q11.2, 16p12.3-16q12.2, 22p13–22q12.3, and 22q12.3–22q13.33) (Fig 1).

### GO analysis and over-representation test

GO analysis was performed using protein analysis through evolutionary relationships (PANTHER) version 14.0 software (http://www.pantherdb.org/), and P-values less than 0.05 were considered statistically significant [20, 21]. In the over-representation test, we used the PANTHER Classification System. Statistical significance calculated by Fisher’s exact test and adjusted using the false discovery rate (FDR) for correction of multiple tests. A FDR-corrected P value threshold of < 0.05 was established.

### PPI network construction and module analysis

A PPI network of genes was constructed with interaction data from STRING, and this was visualized with Cytoscape version 3.8.2 (http://www.cytoscape.org/) [22, 23]. The minimum confidence score was set at 0.700. In order to detect the important modules within the network, ClusterViz based on Molecular Complex Detection (MCODE) tool was used with the following parameters, degree cutoff of 2, node score cutoff of 0.2, k‐core  =  2, and max depth of 100 [24, 25]. This identifies densely connected regions within a network based on topology. The CytoHubba plug-in was used to select the top 10 hub genes within the entire network, according to degree [26]. In addition, the ClueGO plugin was applied for the functional annotation of the top 3 clusters [27].

## Results

Using the UCSC Genome Browser and more particularly, the assembly Dec. 2013 (GRCh38/hg38), we identified 2750 genes in the seven genetic regions (4p14–4q13.3, 5q14.3–5q23.2, 5q23.2–5q34, 15p13–15q11.2, 16p12.3-16q12.2, 22p13–22q12.3, and 22q12.3–22q13.33) (Table 1) where 1305 protein coding genes are located (Table 2).

**Table 1:** Diabetic nephropathy genome scan meta-analysis results.

Bin	Cytogenetic location
Main analysis	
4.3	4p14-4q13.3
5.4	5q14.3-5q23.2
5.5	5q23.2-5q34
15.1	15q11.2-15p13
16.2	16p12.3-16q12.2
22.1	22p13-22q12.3
22.2	22q12.3-22q13.33

**Table 2:** Identification of gene type.

Gene type	BOTH DM
ncRNA	406
other	61
protein coding	1305
pseudo	850
rRNA	1
snoRNA	106
snRNA	3
Total	2732

### GO analysis

To understand the functions of the genes located at these regions, we performed GO analysis using PANTHER version 14.0 software. GO analysis consists of biological process (BP), cellular component (CC), and molecular function (MF) (S1 Table). We chose the top five results based on their percentages (Table 3).

**Table 3:** The top five GO terms per category.

GO Term	Top 5 GO Terms	Percent of gene hit against total # genes
**Molecular Function**		
**1**	binding (GO:0005488)	23.9%
**2**	catalytic activity (GO:0003824)	18.9%
**3**	transporter activity (GO:0005215)	4.9%
**4**	molecular function regulator (GO:0098772)	4.2%
**5**	transcription regulator activity (GO:0140110)	3.7%
**Biological Process**		
**1**	cellular process (GO:0009987)	36.6%
**2**	metabolic process (GO:0008152)	22.8%
**3**	biological regulation (GO:0065007)	21.9%
**4**	response to stimulus (GO:0050896)	13.9%
**5**	cellular component organization or biogenesis (GO:0071840)	11.3%
**Cellular Component**		
**1**	cell (GO:0005623)	44.0%
**2**	cell part (GO:0044464)	44.0%
**3**	organelle (GO:0043226)	27.4%
**4**	membrane (GO:0016020)	17.9%
**5**	protein-containing complex (GO:0032991)	13.3%
**Protein Class**		
**1**	metabolite interconversion enzyme (PC00262)	7.0%
**2**	protein modifying enzyme (PC00260)	5.8%
**3**	nucleic acid binding protein (PC00171)	5.0%
**4**	transporter (PC00227)	4.2%
**5**	gene-specific transcriptional regulator (PC00264)	3.7%
**Panther Pathway**		
**1**	Wnt signaling pathway (P00057)	5.5%
**2**	Cadherin signaling pathway (P00012)	4.4%
**3**	Angiogenesis (P00005)	1.3%
**4**	EGF receptor signaling pathway (P00018)	1.3%
**5**	Gonadotropin-releasing hormone receptor pathway (P06664)	1.2%

**Fig 2:**
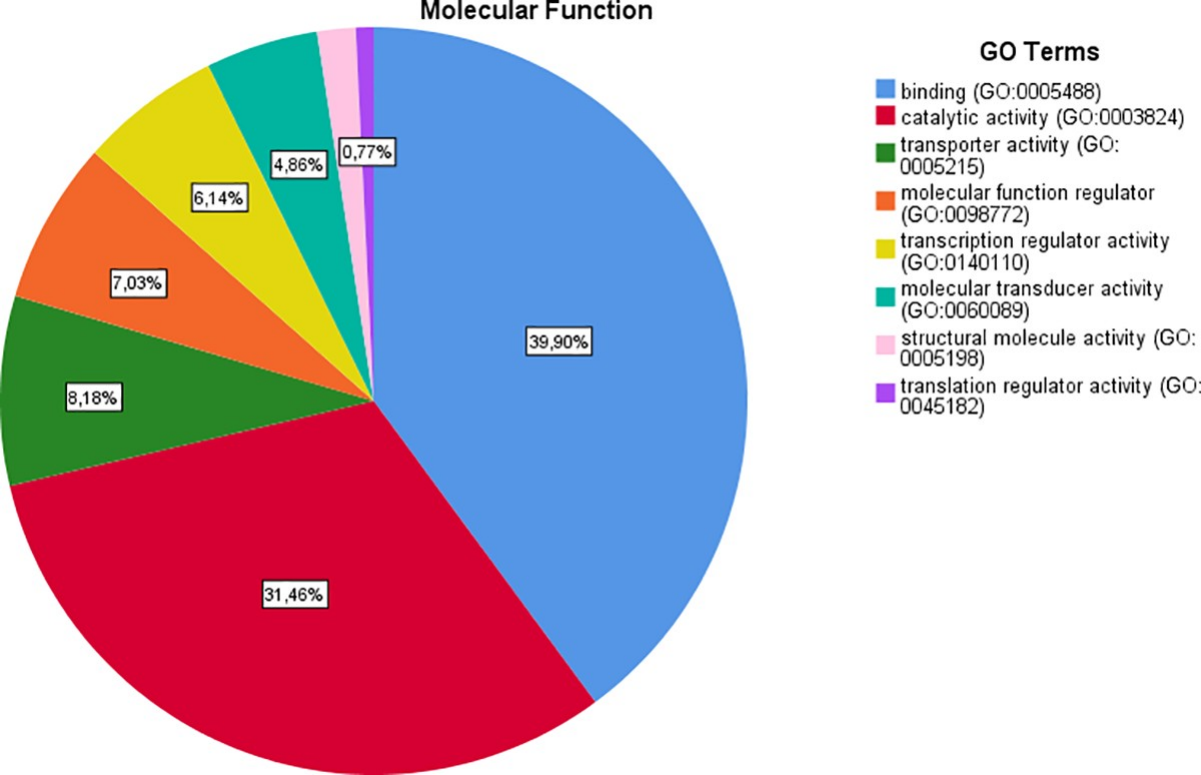
Results of “molecular function” category.

**Fig 3:**
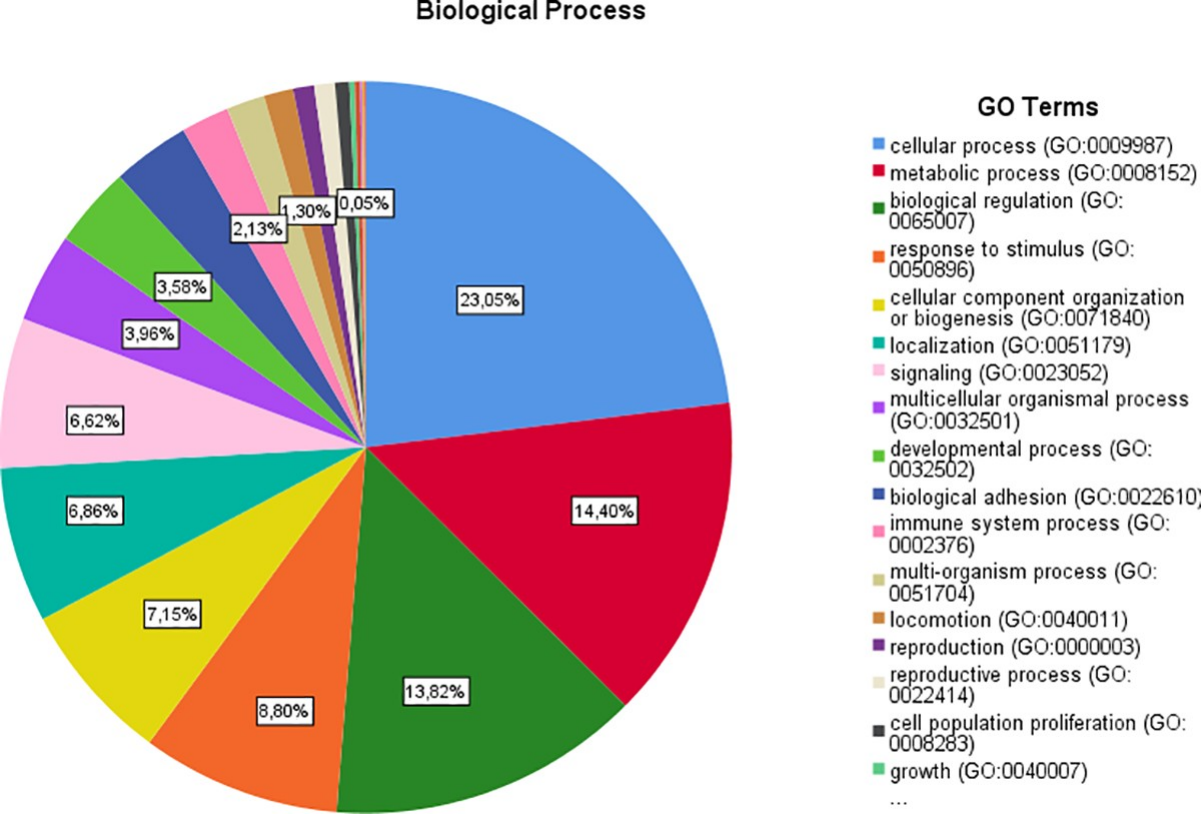
Results of “biological process” category.

**Fig 4:**
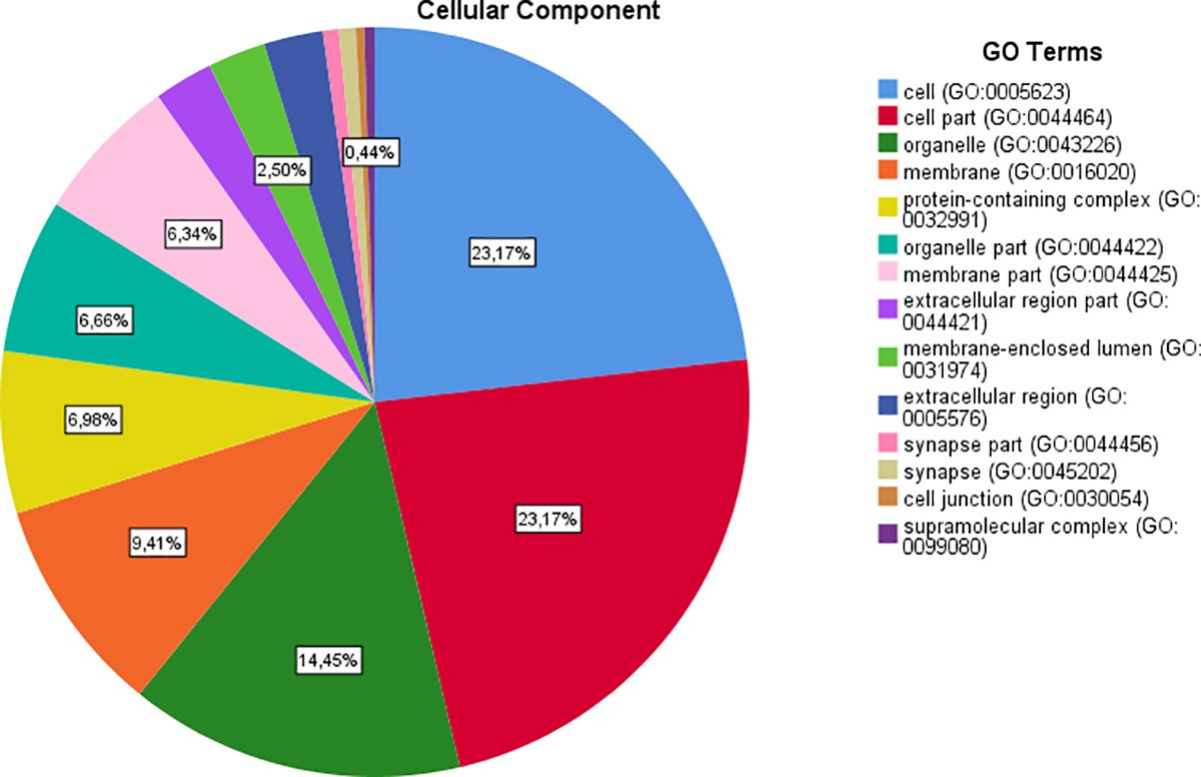
Results of “cellular component” category.

**Fig 5:**
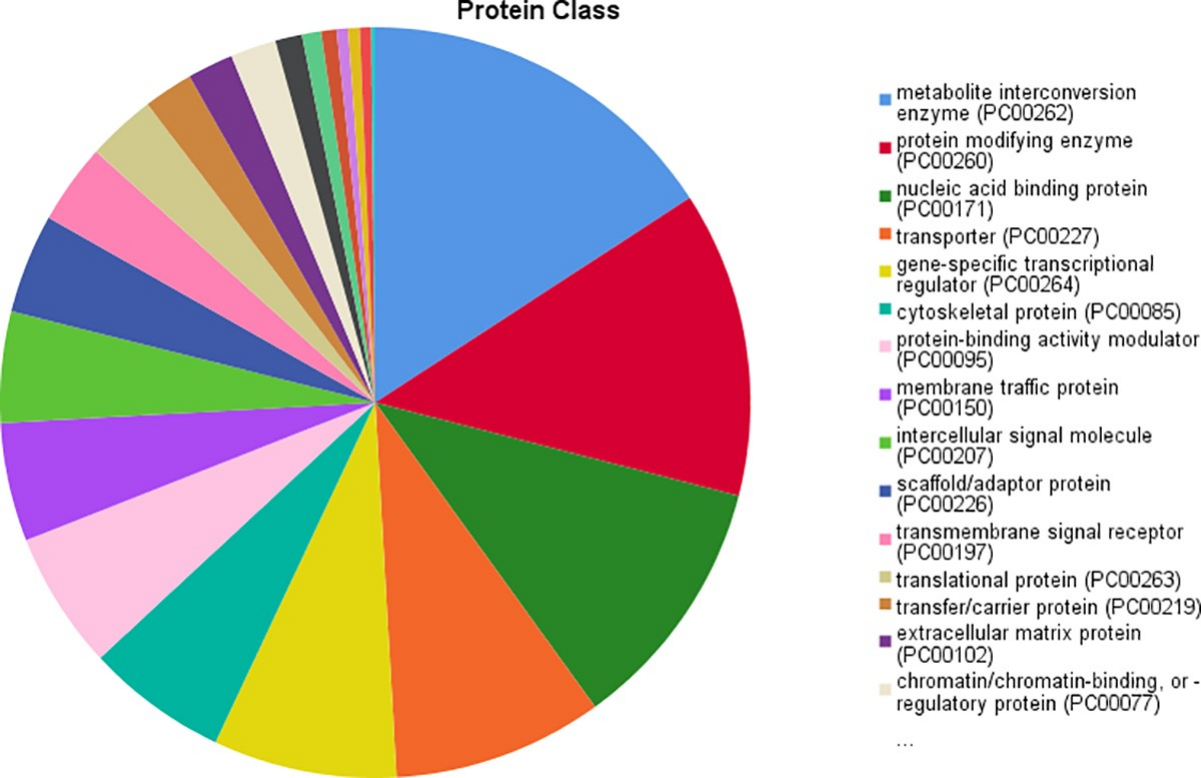
Results of “protein class” category.

**Fig 6:**
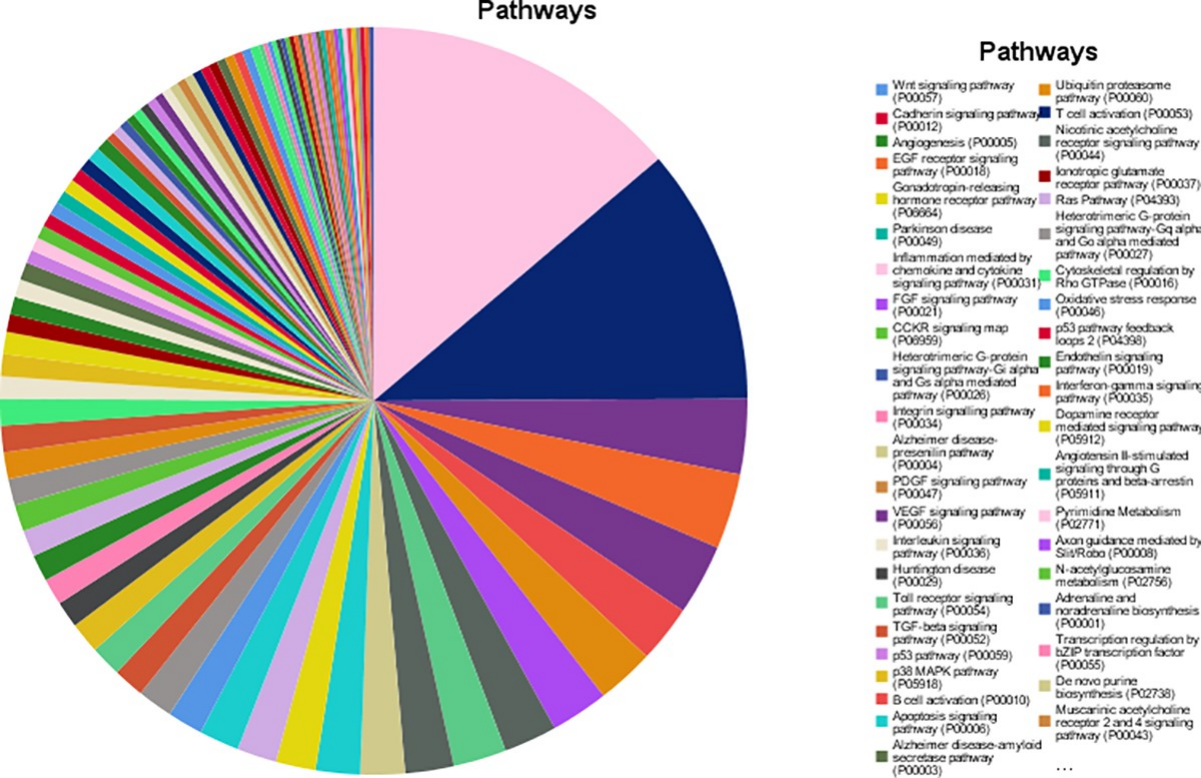
Results of pathway analysis.

Regarding the main meta-analysis genes and the “molecular function” category, it was demonstrated that most of the genes are involved in the binding (23.9% genes), catalytic activity (18.9% genes), transporter activity (4.9% genes), molecular function regulator (4.2% genes) and transcription regulator activity (3.7% genes). With regard to “biological processes” category, the first five GO categories include cellular process (36.6% genes), metabolic process (22.8%), biological regulation (21.9% genes), response to stimulus (13.9% genes) and cellular component organization or biogenesis (11.3% genes). Regarding the “cellular component” category, the majority of the genes were components of the cell (44% genes), cell part (44% genes), organelle (27.4% genes), membrane (17.9% genes) and protein-containing complex (13.3% genes). Regarding the “protein class” category, the most of the proteins are metabolite interconversion enzymes (7% proteins), protein modifying enzymes (5.8% proteins), nucleic acid binding proteins (5% proteins), transporters (4.2% proteins) and gene-specific transcriptional regulators (3.7% proteins). Finally, in the “pathway” category, the majority of genes are involved in Wnt signaling pathway (5.5% pathways), cadherin signaling pathway (4.4% pathways), angiogenesis (1.3% pathways), EGF receptor signaling pathway (1.3% pathways) and gonadotropin-releasing hormone receptor pathway (1.2% pathways) (Table 3) (Figs 2–6).

### Over representation test

Having established the biological functions, protein families and pathways in which genes are involved, we performed over-representation test using PANTHER software to determine which GO terms are statistically significant enriched in our gene list. PANTHER protein class tool compares the set of gene lists to the reference genome, in this case Homo sapiens, and computes if our data set is enriched with categories of gene or protein families (S2 Table). We chose the top five results based on their P-values (Table 4).

**Table 4:** Results of the over-representation test of the main analysis (only over-represented).

	Homo sapiens (REF)	Client Text Box Input (Hierarchy)					
GO biological process complete	#	#	expected	Fold Enrichment	+	raw P value	FDR
homophilic cell adhesion via plasma membrane adhesion molecules	167	59	10.45	5.64	+	7.60E-23	1.21E-18
cell-cell adhesion via plasma-membrane adhesion molecules	256	68	16.02	4.24	+	2.17E-20	1.72E-16
cell-cell adhesion	510	83	31.92	2.60	+	2.69E-13	1.42E-09
calcium-dependent cell-cell adhesion via plasma membrane cell adhesion molecules	42	15	2.63	5.71	+	6.95E-07	9.19E-04
biological adhesion	953	104	59.65	1.74	+	2.12E-07	3.06E-04
**GO molecular function complete**	#	#	expected	Fold Enrichment	+	raw P value	FDR
calcium ion binding	733	95	45.88	2.07	+	3.81E-10	1.82E-06
ligand-gated anion channel activity	19	9	1.19	7.57	+	2.06E-05	8.97E-03
GABA-gated chloride ion channel activity	13	8	.81	9.83	+	1.45E-05	8.64E-03
transferase activity, transferring sulfur-containing groups	73	17	4.57	3.72	+	1.73E-05	8.29E-03
CXCR chemokine receptor binding	18	9	1.13	7.99	+	1.48E-05	7.85E-03
**GO cellular component complete**	#	#	expected	Fold Enrichment	+	raw P value	FDR
integral component of plasma membrane	1656	149	103.64	1.44	+	2.24E-05	4.50E-02
intrinsic component of plasma membrane	1734	154	108.53	1.42	+	2.68E-05	2.69E-02
**PANTHER Protein Class**	#	#	expected	Fold Enrichment	+	raw P value	FDR
chemokine	17	10	1.06	9.40	+	1.63E-06	3.17E-04
cytokine	81	15	5.07	2.96	+	4.76E-04	2.32E-02
**PANTHER Pathways**	#	#	expected	Fold Enrichment	+	raw P value	FDR
Cadherin signaling pathway	160	58	10.01	5.79	+	6.28E-23	1.03E-20
Wnt signaling pathway	317	72	19.84	3.63	+	2.74E-18	2.24E-16

In main analysis, regarding the “biological process” category, the most enriched terms were the homophilic cell adhesion via plasma membrane adhesion molecules, cell-cell adhesion via plasma membrane cell adhesion molecules, cell-cell adhesion, calcium-dependent cell-cell adhesion via plasma membrane cell adhesion molecules and biological adhesion. The most over represented GO terms in the “molecular function” category include the calcium ion binding, ligand-gated anion channel activity, GABA-gated chloride ion channel activity, transferase activity transferring sulfur-containing groups and CXCR chemokine receptor binding. The most enriched GO term in “cellular component” category are integral components of plasma membrane. With regard to the most enriched protein class, the majority of the proteins are chemokines and cytokines, while the most enriched pathways in our gene list are the cadherin and Wnt signaling pathways (Table 4).

### Protein network analysis

**Fig 7:**
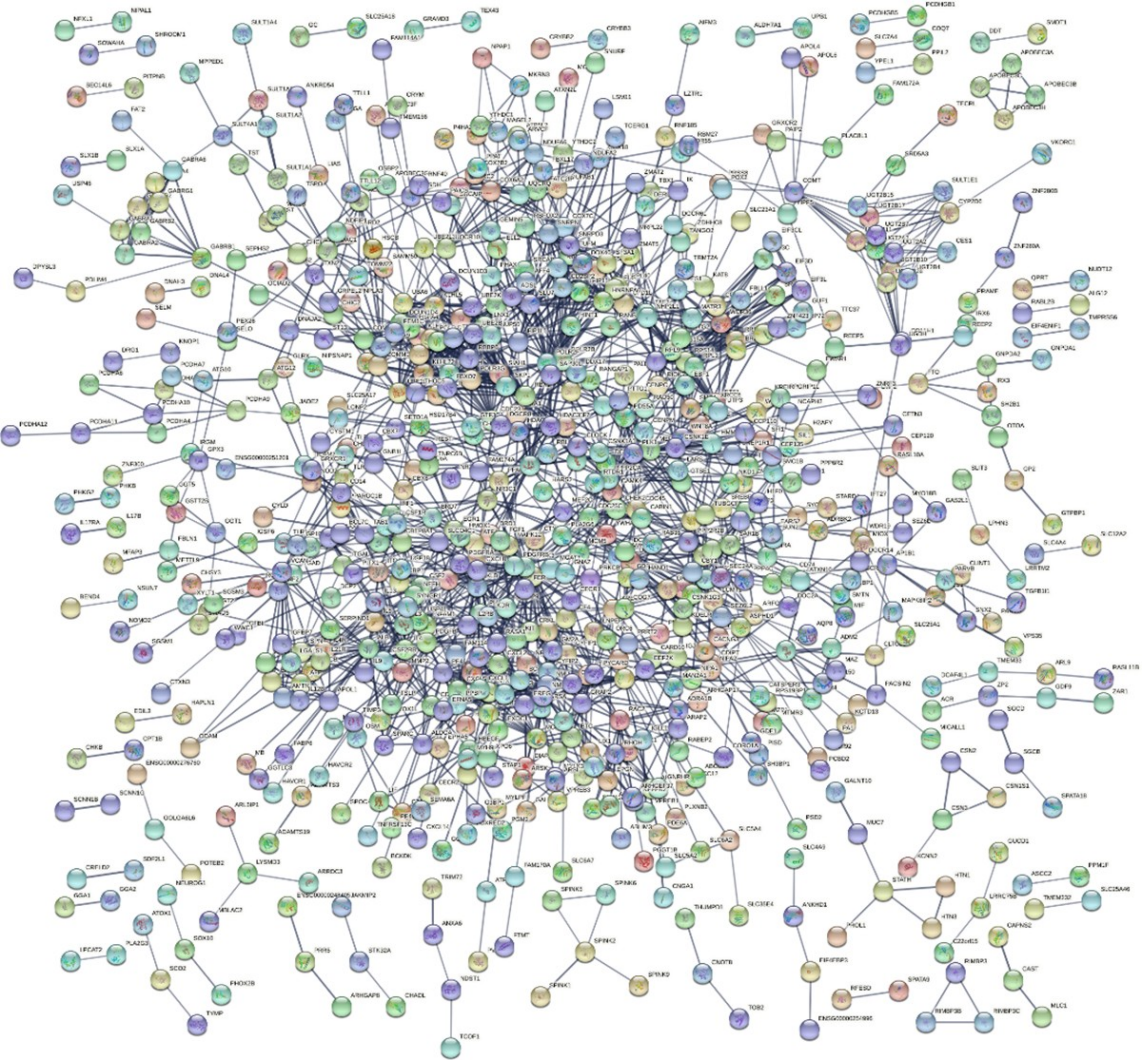
STRING protein network analysis with hidden disconnected nodes in the network. High confidence 0.7, it is full network (the edges indicate both functional and physical protein associations).

For further understanding the function of the 1305 genes harbored in the seven cytogenetic regions, we constructed a PPI network that consists of 1266 nodes and 2047 edges by using STRING database and Cytoscape software (Fig 7). The line thickness indicates the strength of data support. The PPI enrichment p-value is 4.98e-08 that means that this network has significantly more interactions than expected.

**Fig 8:**
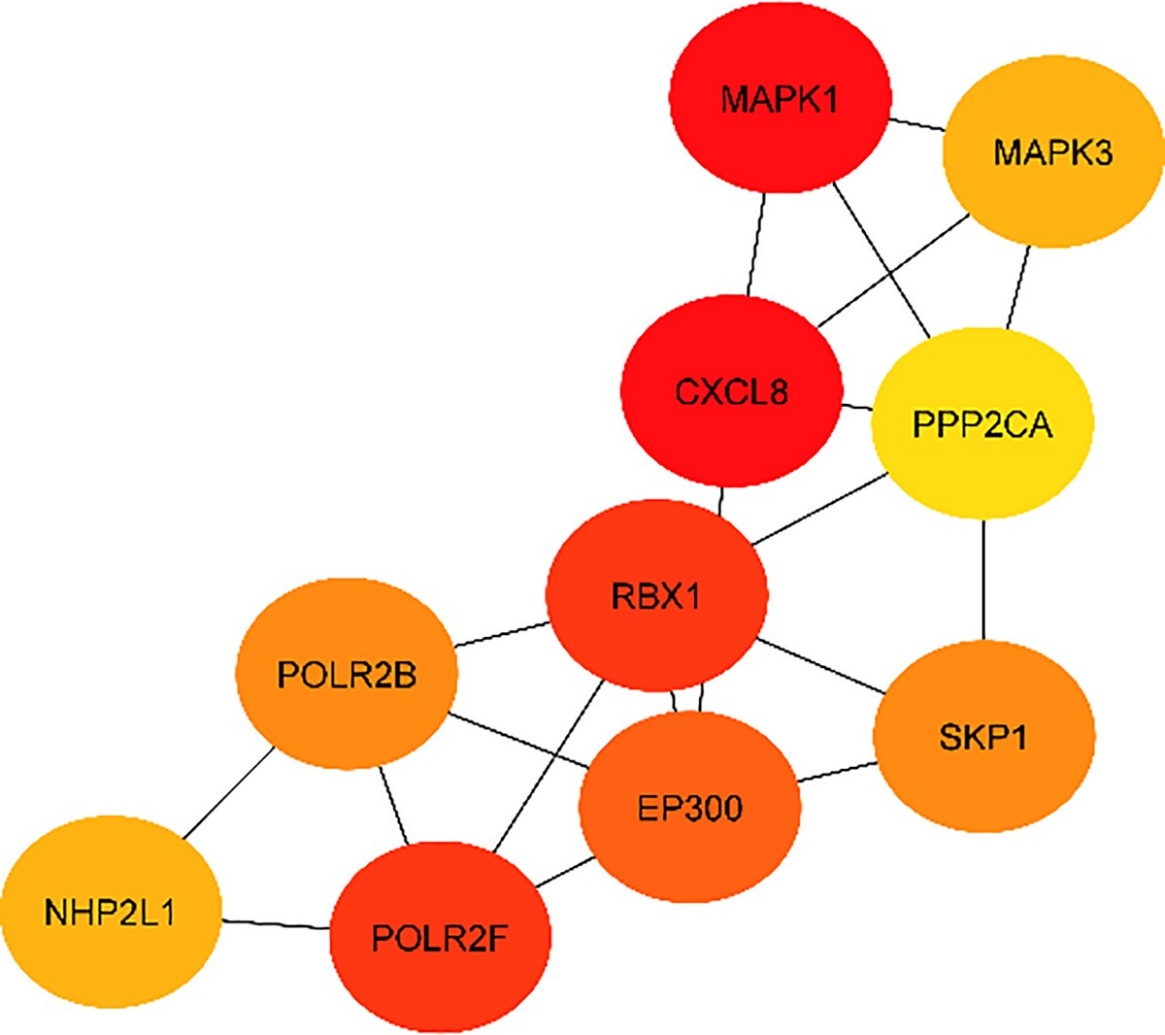
Top 10 nodes based on their degree.

The protein network analysis revealed the following 10 genes as the nodes with the most interactions: *MAPK1*, *CXCL8*, *RBX1*, *POLR2F*, *EP300*, *SKP1*, *POLR2B*, *MAPK3*, *NHP2L1*, *PPP2CA* most of which are enzymes and more specifically kinases, whereas one (*EP300*) is implicated in epigenetic modifications (Table 5) (Fig 8).

**Table 5:** The top 10 nodes based on their degree.

Official gene symbol	Official full name	Degree
*MAPK1*	mitogen-activated protein kinase 1	34
*CXCL8*	C-X-C motif chemokine ligand 8	34
*RBX1*	ring-box 1	33
*POLR2F*	RNA polymerase II, I and III subunit F	33
*EP300*	E1A binding protein p300	31
*SKP1*	S-phase kinase associated protein 1	30
*POLR2B*	RNA polymerase II subunit B	30
*MAPK3*	mitogen-activated protein kinase 3	29
*NHP2L1 (SNU13)*	small nuclear ribonucleoprotein 13	29
*PPP2CA*	protein phosphatase 2 catalytic subunit alpha	28

### MCODE clustering results

**Fig 9:**
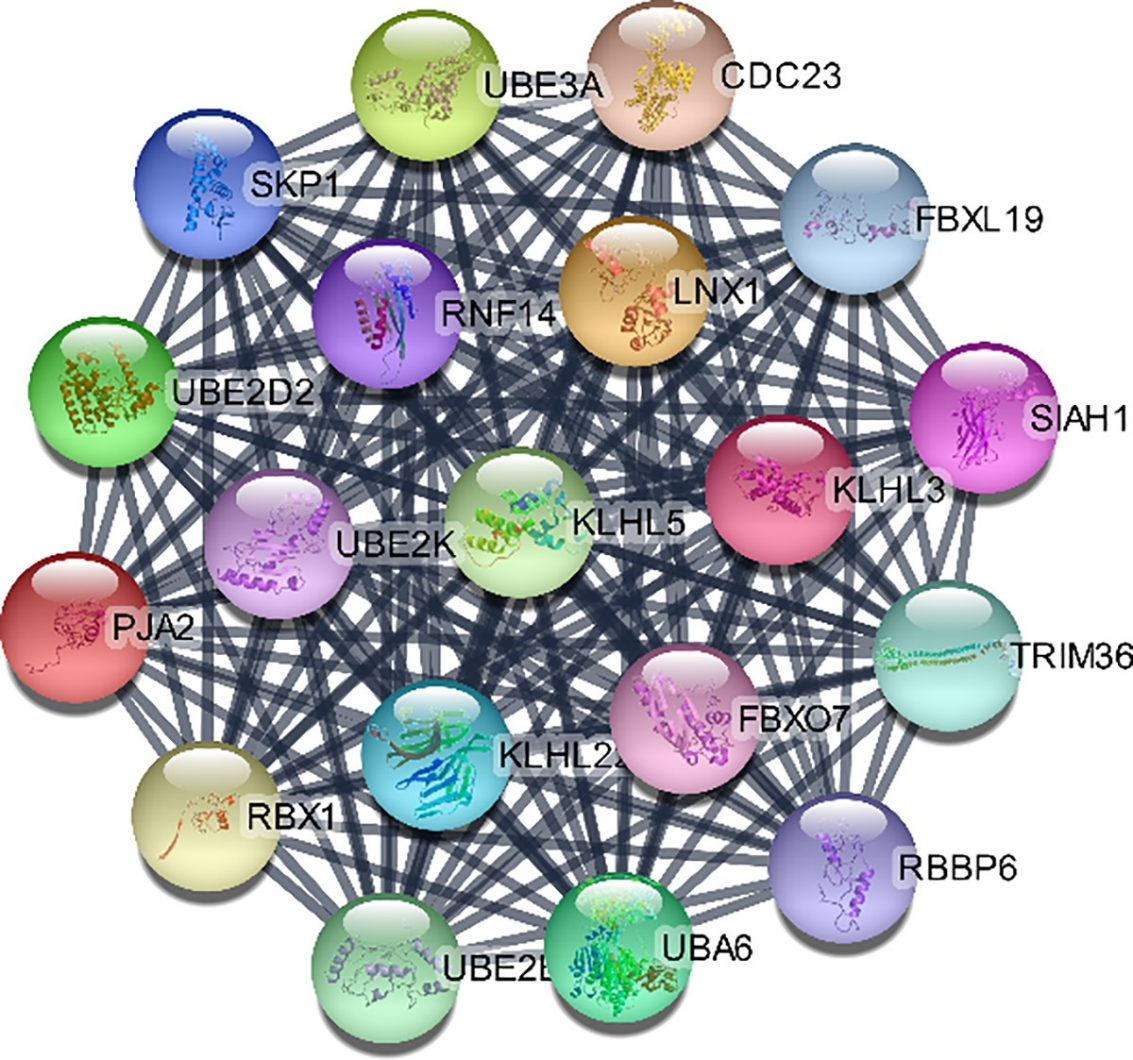
Cluster 1 based on MCODE analysis.

**Fig 10:**
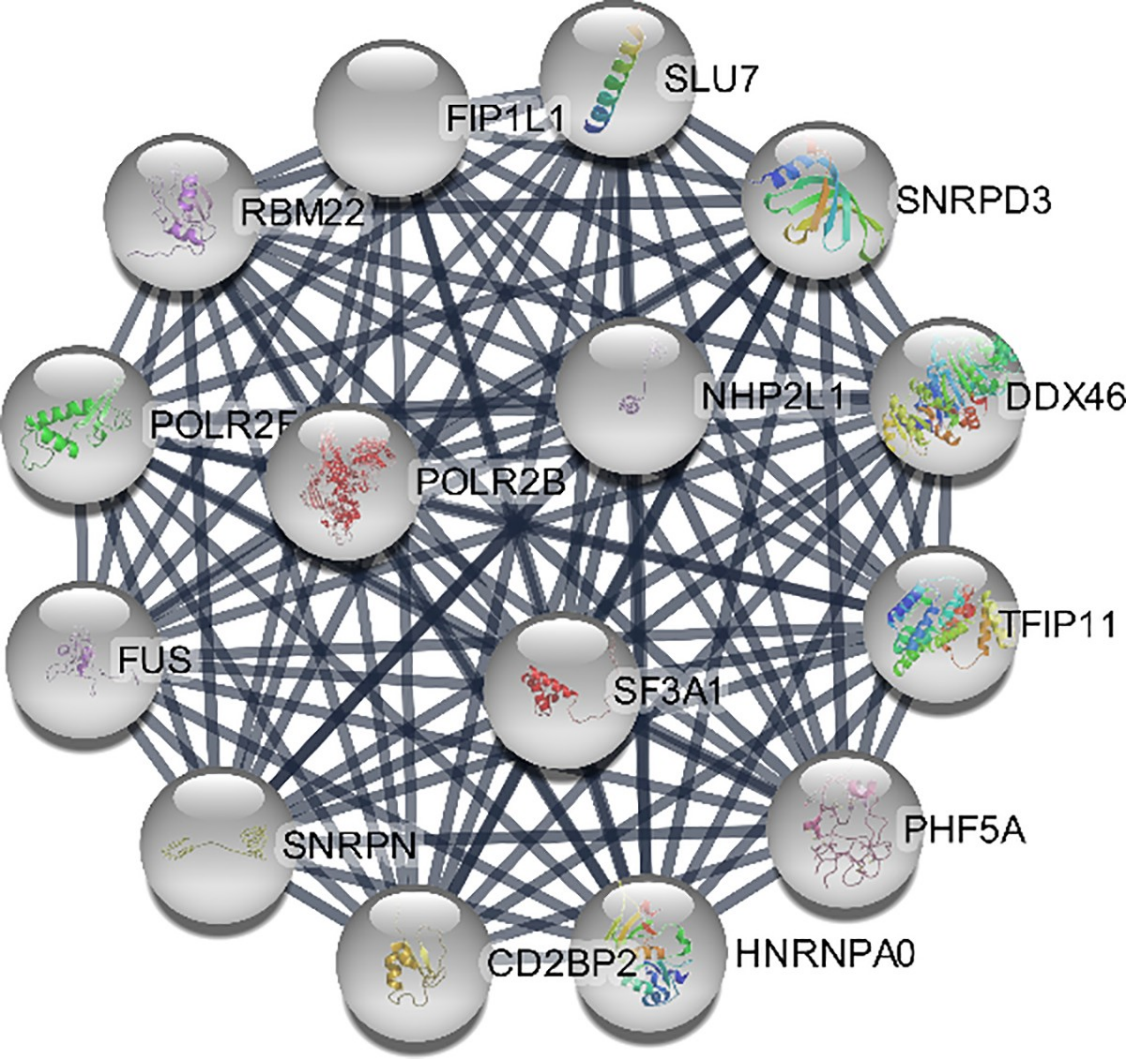
Cluster 2 based on MCODE analysis.

**Fig 11:**
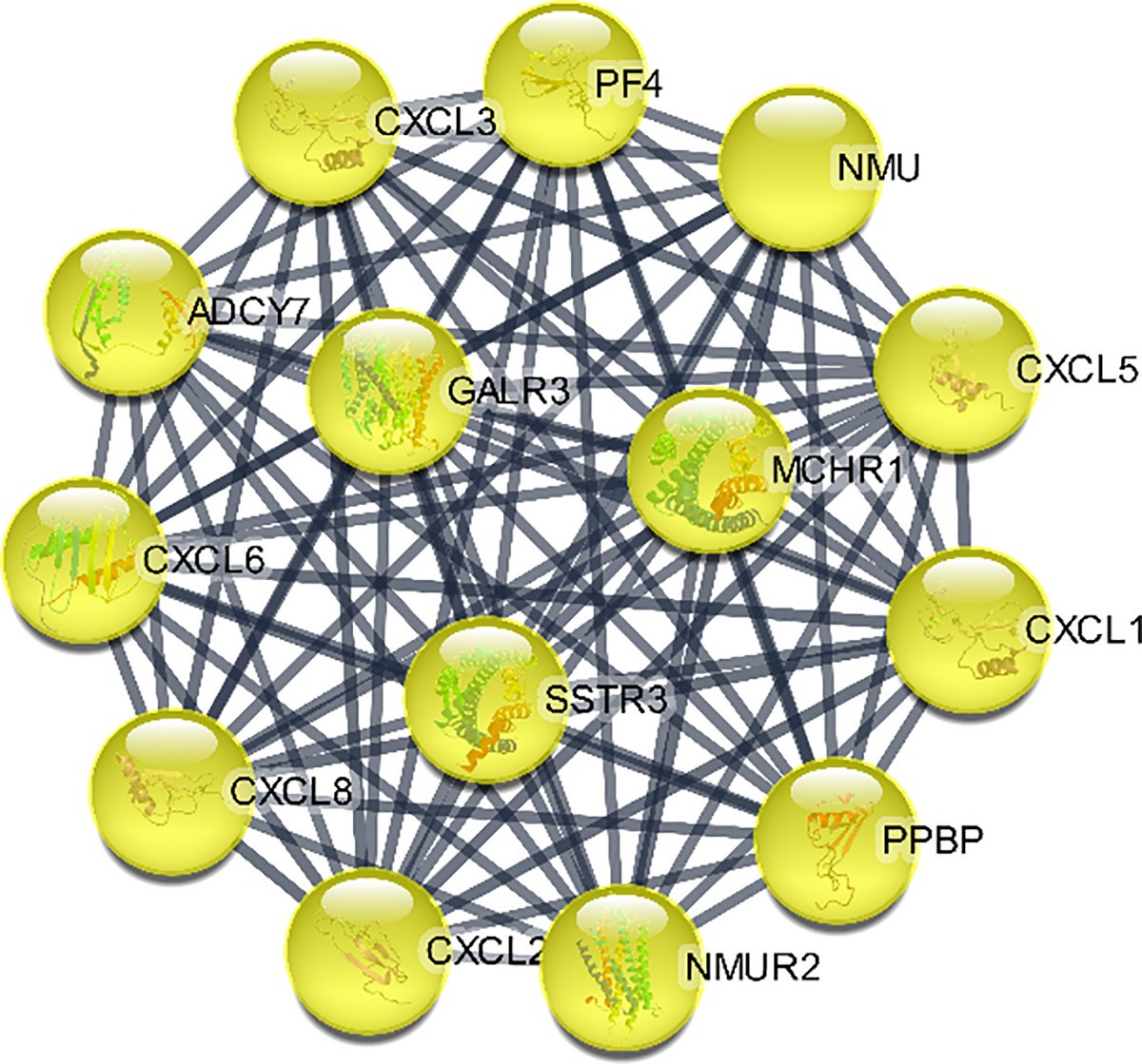
Cluster 3 based on MCODE analysis.

**Fig 12:**
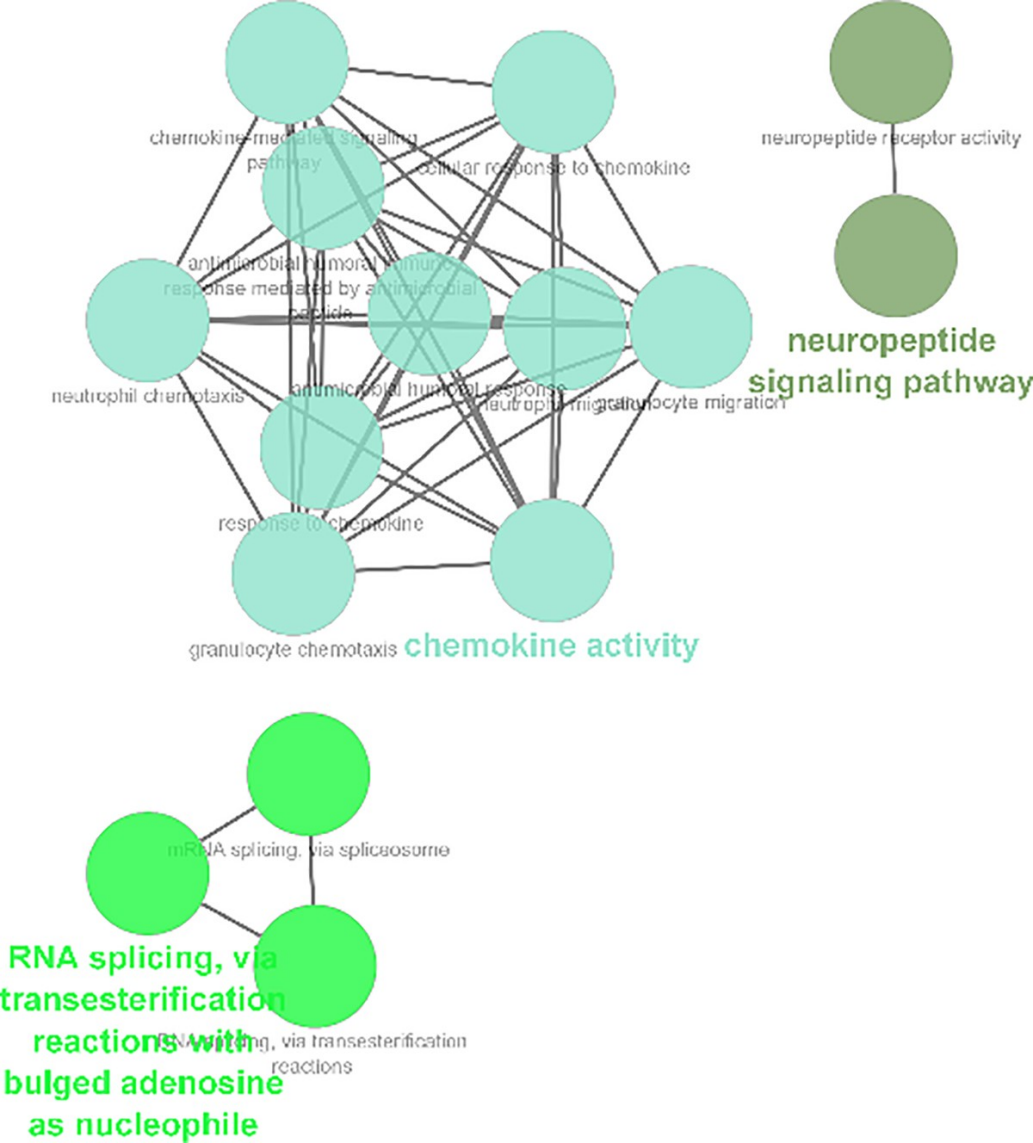
ClueGO annotation results based on biological process analysis.

Moreover, pivotal modules were identified from the PPI network using ClusterViz plugin based on MCODE algorithm in Cytoscape, while ClueGO was used for the functional annotation of the top 3 clusters. Module 1 included 19 nodes with 171 edges (Fig 9) and ClueGO analysis indicated that they were correlated with RNA splicing. Module 2 included 15 nodes with 105 edges significantly enriched (Fig 10) in neuropeptide signaling. Module 3 included 14 nodes with 91 edges related to chemokine signaling pathway (Figs 11 and 12).

## Discussion

In the present study, we used a bioinformatics method to identify key genes and signaling pathways in the diabetic nephropathy pathogenesis with a focus on the role of protein coding genes. A total of 1305 coding genes were located in the seven cytogenetic regions which were identified statistically significant in the meta-analysis of GWLS. Gene ontology with enrichment analysis, pathway analysis and protein network analysis revealed that these genes are involved in specific cellular processes, signaling pathways and gene networks. The data analysis reveals the cell adhesion as the most over-represented biological process among these genes, the calcium ion binding as the most over-represented molecular function and also reveals that integral components of plasma membrane are key regulators in our gene list. Chemokines and cytokines constitute the most significant protein classes, whereas Cadherin and Wnt signaling pathways are the most affected signaling pathways in our gene list.

With regard to the enrichment of chemokines and cytokines, many lines of evidence indicate the role of inflammation and immune response in the pathogenesis of diabetic nephropathy [4, 28]. Although DN is considered as a non-immune disease, proteinuria which is a hallmark of DN, contributes to further tubular and interstitial damage. A recent meta-analysis revealed the significance of variants in *CCL2*, *CCR5*, *IL6*, *IL8*, *EPO*, *IL1A*, *IL1B*, *IL100*, *IL1RN*, *GHRL*, *MMP9*, *TGFB1*, *VEGFA*, *MMP3*, *MMP12*, *IL12RB1*, *PRKCE*, *TNF* and *TNFRSF19* genes with an increased risk of DN [5]. Many studies evaluated altered cytokine expression in DN. For instance, in animal models of DN renal expression of IL-1 is increased [29]. Another clinical study showed that serum levels of IL-6 were substantially higher in patients with DN than in control patients without renal lesions [30]. Patients with DN showed elevated serum levels of IL-18, as well as increased urinary excretion of this cytokine [31]. Various biological effects mediated by TNF are relevant in diabetic nephropathy, including its direct cytotoxicity to renal cells, activation of cell pathways leading to apoptosis and necrosis, and induction of alterations in intraglomerular hemodynamics and reduction of glomerular filtration [32–35]. In addition, TNF is associated with increased endothelial cell permeability [36]. Moreover, many clinical studies have found that both serum and urinary levels of TNF in patients with DN are higher than in nondiabetic individuals, and also higher than in patients with diabetes who have no kidney involvement [4]. Studies in animal models of T1DM and T2DM have shown potential beneficial anti-inflammatory effects on DN with the use of immunosuppressive drugs [4], such as mycophenolate mofetil and infliximab [37, 38].

Regarding the most enriched pathways in our gene list of 1305 coding genes, Cadherin and Wnt signaling pathways, there are findings of convergence between Wnt, β-catenin, and cadherin pathways [39]. Cadherins are glycoproteins that constitute a type of cell adhesion molecules that mediate calcium dependent, homotypic cell–cell adhesion in all solid tissues of the organism[39, 40]. More specifically, E-cadherin which is considered one of the most vital molecules in cell-to-cell adhesion in epithelial tissues, it is localized on the surfaces of epithelial cells in areas of cell-to-cell connection known as adherent’s junctions [41]. A study indicated that levels of E-cadherin decreased before normoalbuminuria concluding that E-cadherin is an early kidney biomarker, a finding that confirms the results of another study which measured urinary soluble E-cadherin and its expression and they demonstrated that the levels significantly raised in the early stage of DN and elevated with the progression of DN [41, 42]. It is also known that human proximal epithelial cells uniquely express N-cadherin instead of E-cadherin as major cell-cell adhesion molecule [43]. Studies have also found that increased levels of urinary protein in DN are associated with podocyte injury, including podocyte apoptosis, detachment and EMT [44]. Altered cadherin expression is implicated in podocyte epithelial-mesenchymal transition (EMT) which is characterized by the loss of epithelial cell markers (e.g., E-cadherin) and re-expression of mesenchymal markers (e.g., vimentin and α-SMA) [45]. These data suggest that altered cadherin expression is involved in DN associated proteinuria.

Wnts are strong regulators of processes like cell proliferation and differentiation, and their signaling pathway involves proteins that participate in both gene transcription and cell adhesion [39]. Proper β-catenin expression is essential to maintain the glomerular filtration barrier and its function [46], whereas several studies have suggested that activation of Wnt/β-catenin signaling promoted podocyte dysfunction in DN [46–48]. It is also known that developmental abnormalities ranging from stem cell loss to kidney and reproductive tract defects are caused by mutations in Wnt genes [49]. In addition, β-catenin is tight ligand to the cytoplasmic part of type I cadherins and is involved in the structural organization and function of cadherins [50, 51].

The findings of the present bioinformatics analysis which found that genes involved in cadherin and Wnt signaling pathways are associated with DN are confirmed and validated by several biological data. Accumulating evidence indicate the involvement of Wnt/β-catenin signaling pathway in renal cell injury including mesangial cells, podocytes [52] and tubular cell damage and also in tubular interstitial fibrosis in DN [46, 53, 54] leading to intense interest about the effects of this pathway in the pathophysiology and progression of DN [55]. Another study found that the levels of β-catenin and WNT proteins were upregulated in the kidney tissues of both Type I and Type ΙΙ Akita mice, streptozotocin-induced diabetic rats and *db/db* mice compared with their non-diabetic controls [54]. However, lowering blood glucose levels by insulin attenuated the activation of WNT signaling pathway [54]. In addition, hyperglycaemia and oxidative stress were found to activate the WNT pathway in the kidneys of diabetic animals [54]. Furthermore, blockade of WNT signaling by a monoclonal antibody to LDL-receptor-related protein 6 (LRP6) ameliorated DN [54] whereas another study found that liraglutide suppressed the production of extracellular matrix proteins and ameliorated renal injury of DN by enhancing Wnt/β-catenin signaling [55]. The aforementioned experimental data suggest the involvement of dysregulated WNT pathway in the diabetic kidney could play a pathogenic role in DN. Zhou et al. also observed a concurrent upregulation of multiple WNT ligands across different diabetic animal models suggesting that most WNT ligands are positively upregulated in the kidneys by diabetes [54]. Moreover, it has been reported an increase of WNT1 protein levels in the podocytes of human kidney biopsies from patients with DN [46]. In addition to DN, obstructive kidney injury and ischaemia-reperfusion injury have also shown to induce overexpression of several WNT ligands and FZD receptors, whereas activation of Wnt/β-catenin was involved in the cyst formation of polycystic kidney disease [56] indicating that WNT signaling pathway could constitute a common pathogenic mechanism of some kidney diseases [54]. Many lines of evidence have also demonstrated that Wnt/*β*-catenin is involved in the epithelial-mesenchymal phenotypic transition of mesangial cells under DN conditions [57], as well as in the apoptotic regulation of mesangial cells [53, 58, 59]. Furthermore, pharmacologic activation of β-catenin induced albuminuria in wild-type mice but not in β-catenin-knockout littermates [46] suggesting that targeting hyperactive Wnt/β-catenin signaling [60] may represent a novel therapeutic strategy for proteinuric kidney diseases and not only for hindering DN [54].

Systems biology approaches in diabetic nephropathy have also indicated Wnt signaling pathway and cytokine-cytokine receptor interaction as significantly related pathways with DN [61]. Other significant pathways include MAPK signaling pathway, extracellular matrix (ECM)-receptor interaction, angiogenesis, PI3-Akt signaling pathway, Jak-STAT signaling pathway, renin-angiotensin pathway, NF-kappa B and TGF-beta signaling pathways, as well as oxidative stress response [61]. Another study also revealed significance of the cytokine-cytokine receptor interaction and Jak-STAT signaling pathway [62]. Systems biology approaches have been already used in chronic kidney disease and other nephrological diseases [63, 64].

In addition, it is noteworthy to be mentioned that non-coding RNAs (ncRNAs) that are located in the seven cytogenetic regions identified from the meta-analysis could further regulate gene expression. Roles of microRNA (miRNA), long ncRNA (lncRNA) and circular RNA (circRNA) in DN have recently studied [65–67]. MiRNA is the best characterized non-coding RNA for transcriptional gene regulation. MiRNAs play significant roles in regulationg inflammation in DN [67]. Regarding circRNAs, they regulate gene expression because they act as sponges of miRNA [68] and play an significant role in renal diseases [69]. Non-coding RNAs as well as other epigenetic modifications, such as DNA methylation and histone modification, modulate numerous inflammatory pathways in DN [65]. Although there are many lines of evidence regarding the roles on non-coding RNAs in DN, further studies are warranted to reveal their specific contribution in the pathogenesis of DN as well as potential therapeutic approaches and diagnostic biomarkers for DN.

## Conclusions

The present study design can decipher the most relevant biological precesses, molecular functions, protein classes and signaling pathways which may point to a novel approach to enhance the understanding of pathophysiology of DN. In conclusion, the cadherin and Wnt signaling pathways might represent promising targets in developing new treatments to prevent not only DN caused by both T1DM and T2DM but a variety of proteinuric kidney diseases in humans and the cytokines and chemokines could also constitute potential therapeutic targets in DN.

## Supporting information

S1 Table Gene ontology analysis results. (DOCX)

S2 Table Protein class analysis. (DOCX)

S3 Table Pathway analysis results. (DOCX)

S1 File Results of the over representation test. (DOCX)
