# Supplementary material for: Cadherin and Wnt signaling pathways as key regulators in diabetic nephropathy
Source: PLoS One. 2021 Aug 19;16(8):e0255728. doi: 10.1371/journal.pone.0255728 (PMC8375992; doi:10.1371/journal.pone.0255728)
Supplement: S1 Table — (DOCX) [file pone.0255728.s001.docx]

S1 Table: Gene ontology analysis results.

| MF | 1 | binding (GO:0005488) | 312 | 23.9% | 39.9% |
| --- | --- | --- | --- | --- | --- |
| MF | 2 | catalytic activity (GO:0003824) | 246 | 18.9% | 31.5% |
| MF | 3 | transporter activity (GO:0005215) | 64 | 4.9% | 8.2% |
| MF | 4 | molecular function regulator (GO:0098772) | 55 | 4.2% | 7,00% |
| MF | 5 | transcription regulator activity (GO:0140110) | 48 | 3.7% | 6.1% |
| MF | 6 | molecular transducer activity (GO:0060089) | 38 | 2.9% | 4.9% |
| MF | 7 | structural molecule activity (GO:0005198) | 13 | 1,00% | 1.7% |
| MF | 8 | translation regulator activity (GO:0045182) | 6 | 0.5% | 0.8% |
| BP | 1 | cellular process (GO:0009987) | 477 | 36.60% | 23.10% |
| BP | 2 | metabolic process (GO:0008152) | 298 | 22.80% | 14.40% |
| BP | 3 | biological regulation (GO:0065007) | 286 | 21.90% | 13.80% |
| BP | 4 | response to stimulus (GO:0050896) | 182 | 13.90% | 8.80% |
| BP | 5 | cellular component organization or biogenesis (GO:0071840) | 148 | 11.30% | 7.20% |
| BP | 6 | localization (GO:0051179) | 142 | 10.90% | 6.90% |
| BP | 7 | signaling (GO:0023052) | 137 | 10.50% | 6.60% |
| BP | 8 | multicellular organismal process (GO:0032501) | 82 | 6.30% | 4.00% |
| BP | 9 | developmental process (GO:0032502) | 74 | 5.70% | 3.60% |
| BP | 10 | biological adhesion (GO:0022610) | 71 | 5.40% | 3.40% |
| BP | 11 | immune system process (GO:0002376) | 44 | 3.40% | 2.10% |
| BP | 12 | multi-organism process (GO:0051704) | 35 | 2.70% | 1.70% |
| BP | 13 | locomotion (GO:0040011) | 27 | 2.10% | 1.30% |
| BP | 14 | reproduction (GO:0000003) | 19 | 1.50% | 0.90% |
| BP | 15 | reproductive process (GO:0022414) | 19 | 1.50% | 0.90% |
| BP | 16 | cell population proliferation (GO:0008283) | 13 | 1.00% | 0.60% |
| BP | 17 | growth (GO:0040007) | 5 | 0.40% | 0.20% |
| BP | 18 | biological phase (GO:0044848) | 5 | 0.40% | 0.20% |
| BP | 19 | biomineralization (GO:0110148) | 2 | 0.20% | 0.10% |
| BP | 20 | rhythmic process (GO:0048511) | 2 | 0.20% | 0.10% |
| BP | 21 | behavior (GO:0007610) | 1 | 0.10% | 0.00% |
| CC | 1 | cell (GO:0005623) | 574 | 44.00% | 23.20% |
| CC | 2 | cell part (GO:0044464) | 574 | 44.00% | 23.20% |
| CC | 3 | organelle (GO:0043226) | 358 | 27.40% | 14.50% |
| CC | 4 | membrane (GO:0016020) | 233 | 17.90% | 9.40% |
| CC | 5 | protein-containing complex (GO:0032991) | 173 | 13.30% | 7.00% |
| CC | 6 | organelle part (GO:0044422) | 165 | 12.60% | 6.70% |
| CC | 7 | membrane part (GO:0044425) | 157 | 12.00% | 6.30% |
| CC | 8 | extracellular region part (GO:0044421) | 63 | 4.80% | 2.50% |
| CC | 9 | membrane-enclosed lumen (GO:0031974) | 62 | 4.80% | 2.50% |
| CC | 10 | extracellular region (GO:0005576) | 63 | 4.80% | 2.50% |
| CC | 11 | synapse part (GO:0044456) | 17 | 1.30% | 0.70% |
| CC | 12 | synapse (GO:0045202) | 18 | 1.40% | 0.70% |
| CC | 13 | cell junction (GO:0030054) | 9 | 0.70% | 0.40% |
| CC | 14 | supramolecular complex (GO:0099080) | 11 | 0.80% | 0.40% |
