# Supplementary material for: Cadherin and Wnt signaling pathways as key regulators in diabetic nephropathy
Source: PLoS One. 2021 Aug 19;16(8):e0255728. doi: 10.1371/journal.pone.0255728 (PMC8375992; doi:10.1371/journal.pone.0255728)
Supplement: S2 Table — (DOCX) [file pone.0255728.s002.docx]

S2 Table: Protein class analysis.

| 1 | metabolite interconversion enzyme (PC00262) | 96 | 7.4% | 15.8% |
| --- | --- | --- | --- | --- |
| 2 | protein modifying enzyme (PC00260) | 80 | 6.1% | 13.2% |
| 3 | nucleic acid binding protein (PC00171) | 67 | 5.1% | 11.00% |
| 4 | transporter (PC00227) | 55 | 4.2% | 9.1% |
| 5 | gene-specific transcriptional regulator (PC00264) | 48 | 3.7% | 7.9% |
| 6 | cytoskeletal protein (PC00085) | 37 | 2.8% | 6.1% |
| 7 | protein-binding activity modulator (PC00095) | 36 | 2.8% | 5.9% |
| 8 | membrane traffic protein (PC00150) | 31 | 2.4% | 5.1% |
| 9 | intercellular signal molecule (PC00207) | 29 | 2.2% | 4.8% |
| 10 | scaffold/adaptor protein (PC00226) | 26 | 2,00% | 4.3% |
| 11 | transmembrane signal receptor (PC00197) | 21 | 1.6% | 3.5% |
| 12 | translational protein (PC00263) | 18 | 1.4% | 3,00% |
| 13 | transfer/carrier protein (PC00219) | 13 | 1.00% | 2.1% |
| 14 | extracellular matrix protein (PC00102) | 12 | 0.9% | 2.00% |
| 15 | chromatin/chromatin-binding, or -regulatory protein (PC00077) | 12 | 0.9% | 2.00% |
| 16 | defense/immunity protein (PC00090) | 7 | 0.5% | 1.2% |
| 17 | cell adhesion molecule (PC00069) | 5 | 0.4% | 0.8% |
| 18 | storage protein (PC00210) | 4 | 0.3% | 0.7% |
| 19 | calcium-binding protein (PC00060) | 3 | 0.2% | 0.5% |
| 20 | chaperone (PC00072) | 3 | 0.2% | 0.5% |
| 21 | structural protein (PC00211) | 3 | 0.2% | 0.5% |
| 22 | cell junction protein (PC00070) | 1 | 0.1% | 0.2% |
