# Supplementary material for: Cadherin and Wnt signaling pathways as key regulators in diabetic nephropathy
Source: PLoS One. 2021 Aug 19;16(8):e0255728. doi: 10.1371/journal.pone.0255728 (PMC8375992; doi:10.1371/journal.pone.0255728)
Supplement: S3 Table — (DOCX) [file pone.0255728.s003.docx]

S3 Table: Pathway analysis results.

| 1 | Wnt signaling pathway (P00057) | 72 | 5.5% | 13.8% |
| --- | --- | --- | --- | --- |
| 2 | Cadherin signaling pathway (P00012) | 58 | 4.4% | 11.1% |
| 3 | Angiogenesis (P00005) | 17 | 1.3% | 3.3% |
| 4 | EGF receptor signaling pathway (P00018) | 17 | 1.3% | 3.3% |
| 5 | Gonadotropin-releasing hormone receptor pathway (P06664) | 16 | 1.2% | 3.1% |
| 6 | Parkinson disease (P00049) | 13 | 1.00% | 2.5% |
| 7 | Inflammation mediated by chemokine and cytokine signaling pathway (P00031) | 13 | 1.00% | 2.5% |
| 8 | FGF signaling pathway (P00021) | 13 | 1.00% | 2.5% |
| 9 | CCKR signaling map (P06959) | 12 | 0.9% | 2.3% |
| 10 | Heterotrimeric G-protein signaling pathway-Gi alpha and Gs alpha mediated pathway (P00026) | 12 | 0.9% | 2.3% |
| 11 | Integrin signalling pathway (P00034) | 11 | 0.8% | 2.1% |
| 12 | Alzheimer disease-presenilin pathway (P00004) | 10 | 0.8% | 1.9% |
| 13 | PDGF signaling pathway (P00047) | 10 | 0.8% | 1.9% |
| 14 | VEGF signaling pathway (P00056) | 9 | 0.7% | 1.7% |
| 15 | Interleukin signaling pathway (P00036) | 9 | 0.7% | 1.7% |
| 16 | Huntington disease (P00029) | 9 | 0.7% | 1.7% |
| 17 | Toll receptor signaling pathway (P00054) | 8 | 0.6% | 1.5% |
| 18 | TGF-beta signaling pathway (P00052) | 8 | 0.6% | 1.5% |
| 19 | p53 pathway (P00059) | 7 | 0.5% | 1.3% |
| 20 | p38 MAPK pathway (P05918) | 7 | 0.5% | 1.3% |
| 21 | B cell activation (P00010) | 7 | 0.5% | 1.3% |
| 22 | Apoptosis signaling pathway (P00006) | 6 | 0.5% | 1.1% |
| 23 | Alzheimer disease-amyloid secretase pathway (P00003) | 6 | 0.5% | 1.1% |
| 24 | Ubiquitin proteasome pathway (P00060) | 6 | 0.5% | 1.1% |
| 25 | T cell activation (P00053) | 6 | 0.5% | 1.1% |
| 26 | Nicotinic acetylcholine receptor signaling pathway (P00044) | 6 | 0.5% | 1.1% |
| 27 | Ionotropic glutamate receptor pathway (P00037) | 6 | 0.5% | 1.1% |
| 28 | Ras Pathway (P04393) | 6 | 0.5% | 1.1% |
| 29 | Heterotrimeric G-protein signaling pathway-Gq alpha and Go alpha mediated pathway (P00027) | 6 | 0.5% | 1.1% |
| 30 | Cytoskeletal regulation by Rho GTPase (P00016) | 6 | 0.5% | 1.1% |
| 31 | Oxidative stress response (P00046) | 5 | 0.4% | 1,00% |
| 32 | p53 pathway feedback loops 2 (P04398) | 5 | 0.4% | 1,00% |
| 33 | Endothelin signaling pathway (P00019) | 5 | 0.4% | 1,00% |
| 34 | Interferon-gamma signaling pathway (P00035) | 4 | 0.3% | 0.8% |
| 35 | Dopamine receptor mediated signaling pathway (P05912) | 4 | 0.3% | 0.8% |
| 36 | Angiotensin II-stimulated signaling through G proteins and beta-arrestin (P05911) | 4 | 0.3% | 0.8% |
| 37 | Pyrimidine Metabolism (P02771) | 4 | 0.3% | 0.8% |
| 38 | Axon guidance mediated by Slit/Robo (P00008) | 3 | 0.2% | 0.6% |
| 39 | N-acetylglucosamine metabolism (P02756) | 3 | 0.2% | 0.6% |
| 40 | Adrenaline and noradrenaline biosynthesis (P00001) | 3 | 0.2% | 0.6% |
| 41 | Transcription regulation by bZIP transcription factor (P00055) | 3 | 0.2% | 0.6% |
| 42 | De novo purine biosynthesis (P02738) | 3 | 0.2% | 0.6% |
| 43 | Muscarinic acetylcholine receptor 2 and 4 signaling pathway (P00043) | 3 | 0.2% | 0.6% |
| 44 | Muscarinic acetylcholine receptor 1 and 3 signaling pathway (P00042) | 3 | 0.2% | 0.6% |
| 45 | Metabotropic glutamate receptor group III pathway (P00039) | 3 | 0.2% | 0.6% |
| 46 | Insulin/IGF pathway-mitogen activated protein kinase kinase/MAP kinase cascade (P00032) | 3 | 0.2% | 0.6% |
| 47 | Beta2 adrenergic receptor signaling pathway (P04378) | 3 | 0.2% | 0.6% |
| 48 | 5HT4 type receptor mediated signaling pathway (P04376) | 3 | 0.2% | 0.6% |
| 49 | Axon guidance mediated by semaphorins (P00007) | 2 | 0.2% | 0.4% |
| 50 | Coenzyme A biosynthesis (P02736) | 2 | 0.2% | 0.4% |
| 51 | Notch signaling pathway (P00045) | 2 | 0.2% | 0.4% |
| 52 | Metabotropic glutamate receptor group II pathway (P00040) | 2 | 0.2% | 0.4% |
| 53 | Thyrotropin-releasing hormone receptor signaling pathway (P04394) | 2 | 0.2% | 0.4% |
| 54 | Oxytocin receptor mediated signaling pathway (P04391) | 2 | 0.2% | 0.4% |
| 55 | Heterotrimeric G-protein signaling pathway-rod outer segment phototransduction (P00028) | 2 | 0.2% | 0.4% |
| 56 | Opioid proopiomelanocortin pathway (P05917) | 2 | 0.2% | 0.4% |
| 57 | Hedgehog signaling pathway (P00025) | 2 | 0.2% | 0.4% |
| 58 | Opioid prodynorphin pathway (P05916) | 2 | 0.2% | 0.4% |
| 59 | Opioid proenkephalin pathway (P05915) | 2 | 0.2% | 0.4% |
| 60 | General transcription regulation (P00023) | 2 | 0.2% | 0.4% |
| 61 | Salvage pyrimidine deoxyribonucleotides (P02774) | 2 | 0.2% | 0.4% |
| 62 | DNA replication (P00017) | 2 | 0.2% | 0.4% |
| 63 | Circadian clock system (P00015) | 2 | 0.2% | 0.4% |
| 64 | Beta3 adrenergic receptor signaling pathway (P04379) | 2 | 0.2% | 0.4% |
| 65 | Blood coagulation (P00011) | 2 | 0.2% | 0.4% |
| 66 | Beta1 adrenergic receptor signaling pathway (P04377) | 2 | 0.2% | 0.4% |
| 67 | 5HT2 type receptor mediated signaling pathway (P04374) | 2 | 0.2% | 0.4% |
| 68 | 5HT1 type receptor mediated signaling pathway (P04373) | 2 | 0.2% | 0.4% |
| 69 | Axon guidance mediated by netrin (P00009) | 1 | 0.1% | 0.2% |
| 70 | O-antigen biosynthesis (P02757) | 1 | 0.1% | 0.2% |
| 71 | Alpha adrenergic receptor signaling pathway (P00002) | 1 | 0.1% | 0.2% |
| 72 | Mannose metabolism (P02752) | 1 | 0.1% | 0.2% |
| 73 | Lipoate_biosynthesis (P02750) | 1 | 0.1% | 0.2% |
| 74 | Heme biosynthesis (P02746) | 1 | 0.1% | 0.2% |
| 75 | Fructose galactose metabolism (P02744) | 1 | 0.1% | 0.2% |
| 76 | Formyltetrahydroformate biosynthesis (P02743) | 1 | 0.1% | 0.2% |
| 77 | TCA cycle (P00051) | 1 | 0.1% | 0.2% |
| 78 | Metabotropic glutamate receptor group I pathway (P00041) | 1 | 0.1% | 0.2% |
| 79 | Synaptic vesicle trafficking (P05734) | 1 | 0.1% | 0.2% |
| 80 | GABA-B receptor II signaling (P05731) | 1 | 0.1% | 0.2% |
| 81 | Androgen/estrogene/progesterone biosynthesis (P02727) | 1 | 0.1% | 0.2% |
| 82 | p53 pathway by glucose deprivation (P04397) | 1 | 0.1% | 0.2% |
| 83 | Hypoxia response via HIF activation (P00030) | 1 | 0.1% | 0.2% |
| 84 | Vitamin D metabolism and pathway (P04396) | 1 | 0.1% | 0.2% |
| 85 | Vasopressin synthesis (P04395) | 1 | 0.1% | 0.2% |
| 86 | Glycolysis (P00024) | 1 | 0.1% | 0.2% |
| 87 | Nicotine degradation (P05914) | 1 | 0.1% | 0.2% |
| 88 | Enkephalin release (P05913) | 1 | 0.1% | 0.2% |
| 89 | FAS signaling pathway (P00020) | 1 | 0.1% | 0.2% |
| 90 | Histamine H2 receptor mediated signaling pathway (P04386) | 1 | 0.1% | 0.2% |
| 91 | Histamine H1 receptor mediated signaling pathway (P04385) | 1 | 0.1% | 0.2% |
| 92 | Cortocotropin releasing factor receptor signaling pathway (P04380) | 1 | 0.1% | 0.2% |
| 93 | 5HT3 type receptor mediated signaling pathway (P04375) | 1 | 0.1% | 0.2% |
| 94 | 5-Hydroxytryptamine degredation (P04372) | 1 | 0.1% | 0.2% |
