# Supplementary material for: Cadherin and Wnt signaling pathways as key regulators in diabetic nephropathy
Source: PLoS One. 2021 Aug 19;16(8):e0255728. doi: 10.1371/journal.pone.0255728 (PMC8375992; doi:10.1371/journal.pone.0255728)
Supplement: S1 File — (DOCX) [file pone.0255728.s004.docx]

**S1 File.** Results of the over representation test.

|  | [Homo sapiens](http://pantherdb.org/tools/gxIdsList.do?reflist=1) (REF) | [Client Text Box Input](http://pantherdb.org/tools/gxIdsList.do?list=Client%20Text%20Box%20Input&organism=Homo%20sapiens) ([Hierarchy](http://pantherdb.org/tools/compareToRefList.jsp?sortOrder=1&sortList=Client%20Text%20Box%20Input)) NEW! [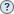](javascript:;)) | | | | | |
| --- | --- | --- | --- | --- | --- | --- | --- |
| [**GO biological process complete**](http://pantherdb.org/tools/compareToRefList.jsp?sortOrder=1&sortList=categories) | [#](http://pantherdb.org/tools/compareToRefList.jsp?sortOrder=2&sortList=Homo%20sapiens) | [#](http://pantherdb.org/tools/compareToRefList.jsp?sortOrder=2&sortList=Client%20Text%20Box%20Input&sortField=num) | [expected](http://pantherdb.org/tools/compareToRefList.jsp?sortOrder=2&sortList=Client%20Text%20Box%20Input&sortField=exp) | [Fold Enrichment](http://pantherdb.org/tools/compareToRefList.jsp?sortOrder=2&sortList=Client%20Text%20Box%20Input&sortField=foldEnrich) | [+/-](http://pantherdb.org/tools/compareToRefList.jsp?sortOrder=1&sortList=Client%20Text%20Box%20Input&sortField=rep) | [raw P value](http://pantherdb.org/tools/compareToRefList.jsp?sortOrder=1&sortList=Client%20Text%20Box%20Input&sortField=pval) | 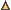 [FDR](http://pantherdb.org/tools/compareToRefList.jsp?sortOrder=2&sortList=Client%20Text%20Box%20Input&sortField=fdr) |
| [homophilic cell adhesion via plasma membrane adhesion molecules](http://amigo.geneontology.org/amigo/term/GO:0007156) | [167](http://pantherdb.org/tools/gxIdsList.do?acc=GO:0007156&reflist=1) | [59](http://pantherdb.org/tools/gxIdsList.do?acc=GO:0007156&list=Client%20Text%20Box%20Input&organism=Homo%20sapiens) | 10.45 | 5.64 | + | 7.60E-23 | 1.21E-18 |
| [cell-cell adhesion via plasma-membrane adhesion molecules](http://amigo.geneontology.org/amigo/term/GO:0098742) | [256](http://pantherdb.org/tools/gxIdsList.do?acc=GO:0098742&reflist=1) | [68](http://pantherdb.org/tools/gxIdsList.do?acc=GO:0098742&list=Client%20Text%20Box%20Input&organism=Homo%20sapiens) | 16.02 | 4.24 | + | 2.17E-20 | 1.72E-16 |
| [cell-cell adhesion](http://amigo.geneontology.org/amigo/term/GO:0098609) | [510](http://pantherdb.org/tools/gxIdsList.do?acc=GO:0098609&reflist=1) | [83](http://pantherdb.org/tools/gxIdsList.do?acc=GO:0098609&list=Client%20Text%20Box%20Input&organism=Homo%20sapiens) | 31.92 | 2.60 | + | 2.69E-13 | 1.42E-09 |
| [detection of chemical stimulus involved in sensory perception](http://amigo.geneontology.org/amigo/term/GO:0050907) | [485](http://pantherdb.org/tools/gxIdsList.do?acc=GO:0050907&reflist=1) | [3](http://pantherdb.org/tools/gxIdsList.do?acc=GO:0050907&list=Client%20Text%20Box%20Input&organism=Homo%20sapiens) | 30.35 | .10 | - | 1.14E-09 | 4.53E-06 |
| [detection of chemical stimulus involved in sensory perception of smell](http://amigo.geneontology.org/amigo/term/GO:0050911) | [440](http://pantherdb.org/tools/gxIdsList.do?acc=GO:0050911&reflist=1) | [3](http://pantherdb.org/tools/gxIdsList.do?acc=GO:0050911&list=Client%20Text%20Box%20Input&organism=Homo%20sapiens) | 27.54 | .11 | - | 1.62E-08 | 5.14E-05 |
| [sensory perception of smell](http://amigo.geneontology.org/amigo/term/GO:0007608) | [466](http://pantherdb.org/tools/gxIdsList.do?acc=GO:0007608&reflist=1) | [4](http://pantherdb.org/tools/gxIdsList.do?acc=GO:0007608&list=Client%20Text%20Box%20Input&organism=Homo%20sapiens) | 29.17 | .14 | - | 2.52E-08 | 6.66E-05 |
| [detection of chemical stimulus](http://amigo.geneontology.org/amigo/term/GO:0009593) | [521](http://pantherdb.org/tools/gxIdsList.do?acc=GO:0009593&reflist=1) | [6](http://pantherdb.org/tools/gxIdsList.do?acc=GO:0009593&list=Client%20Text%20Box%20Input&organism=Homo%20sapiens) | 32.61 | .18 | - | 3.42E-08 | 7.74E-05 |
| [detection of stimulus involved in sensory perception](http://amigo.geneontology.org/amigo/term/GO:0050906) | [550](http://pantherdb.org/tools/gxIdsList.do?acc=GO:0050906&reflist=1) | [7](http://pantherdb.org/tools/gxIdsList.do?acc=GO:0050906&list=Client%20Text%20Box%20Input&organism=Homo%20sapiens) | 34.42 | .20 | - | 4.49E-08 | 8.91E-05 |
| [sensory perception of chemical stimulus](http://amigo.geneontology.org/amigo/term/GO:0007606) | [538](http://pantherdb.org/tools/gxIdsList.do?acc=GO:0007606&reflist=1) | [7](http://pantherdb.org/tools/gxIdsList.do?acc=GO:0007606&list=Client%20Text%20Box%20Input&organism=Homo%20sapiens) | 33.67 | .21 | - | 9.09E-08 | 1.60E-04 |
| [cell adhesion](http://amigo.geneontology.org/amigo/term/GO:0007155) | [947](http://pantherdb.org/tools/gxIdsList.do?acc=GO:0007155&reflist=1) | [104](http://pantherdb.org/tools/gxIdsList.do?acc=GO:0007155&list=Client%20Text%20Box%20Input&organism=Homo%20sapiens) | 59.27 | 1.75 | + | 1.86E-07 | 2.94E-04 |
| [biological adhesion](http://amigo.geneontology.org/amigo/term/GO:0022610) | [953](http://pantherdb.org/tools/gxIdsList.do?acc=GO:0022610&reflist=1) | [104](http://pantherdb.org/tools/gxIdsList.do?acc=GO:0022610&list=Client%20Text%20Box%20Input&organism=Homo%20sapiens) | 59.65 | 1.74 | + | 2.12E-07 | 3.06E-04 |
| [calcium-dependent cell-cell adhesion via plasma membrane cell adhesion molecules](http://amigo.geneontology.org/amigo/term/GO:0016339) | [42](http://pantherdb.org/tools/gxIdsList.do?acc=GO:0016339&reflist=1) | [15](http://pantherdb.org/tools/gxIdsList.do?acc=GO:0016339&list=Client%20Text%20Box%20Input&organism=Homo%20sapiens) | 2.63 | 5.71 | + | 6.95E-07 | 9.19E-04 |
| [synapse organization](http://amigo.geneontology.org/amigo/term/GO:0050808) | [273](http://pantherdb.org/tools/gxIdsList.do?acc=GO:0050808&reflist=1) | [39](http://pantherdb.org/tools/gxIdsList.do?acc=GO:0050808&list=Client%20Text%20Box%20Input&organism=Homo%20sapiens) | 17.09 | 2.28 | + | 8.76E-06 | 1.07E-02 |
| [cytidine to uridine editing](http://amigo.geneontology.org/amigo/term/GO:0016554) | [12](http://pantherdb.org/tools/gxIdsList.do?acc=GO:0016554&reflist=1) | [8](http://pantherdb.org/tools/gxIdsList.do?acc=GO:0016554&list=Client%20Text%20Box%20Input&organism=Homo%20sapiens) | .75 | 10.65 | + | 9.44E-06 | 1.07E-02 |
| [regulation of single stranded viral RNA replication via double stranded DNA intermediate](http://amigo.geneontology.org/amigo/term/GO:0045091) | [19](http://pantherdb.org/tools/gxIdsList.do?acc=GO:0045091&reflist=1) | [9](http://pantherdb.org/tools/gxIdsList.do?acc=GO:0045091&list=Client%20Text%20Box%20Input&organism=Homo%20sapiens) | 1.19 | 7.57 | + | 2.06E-05 | 2.18E-02 |
| [synapse assembly](http://amigo.geneontology.org/amigo/term/GO:0007416) | [100](http://pantherdb.org/tools/gxIdsList.do?acc=GO:0007416&reflist=1) | [20](http://pantherdb.org/tools/gxIdsList.do?acc=GO:0007416&list=Client%20Text%20Box%20Input&organism=Homo%20sapiens) | 6.26 | 3.20 | + | 2.27E-05 | 2.25E-02 |
| [DNA cytosine deamination](http://amigo.geneontology.org/amigo/term/GO:0070383) | [10](http://pantherdb.org/tools/gxIdsList.do?acc=GO:0070383&reflist=1) | [7](http://pantherdb.org/tools/gxIdsList.do?acc=GO:0070383&list=Client%20Text%20Box%20Input&organism=Homo%20sapiens) | .63 | 11.18 | + | 2.79E-05 | 2.60E-02 |
| [negative regulation of single stranded viral RNA replication via double stranded DNA intermediate](http://amigo.geneontology.org/amigo/term/GO:0045869) | [16](http://pantherdb.org/tools/gxIdsList.do?acc=GO:0045869&reflist=1) | [8](http://pantherdb.org/tools/gxIdsList.do?acc=GO:0045869&list=Client%20Text%20Box%20Input&organism=Homo%20sapiens) | 1.00 | 7.99 | + | 4.46E-05 | 3.93E-02 |
| [sulfation](http://amigo.geneontology.org/amigo/term/GO:0051923) | [23](http://pantherdb.org/tools/gxIdsList.do?acc=GO:0051923&reflist=1) | [9](http://pantherdb.org/tools/gxIdsList.do?acc=GO:0051923&list=Client%20Text%20Box%20Input&organism=Homo%20sapiens) | 1.44 | 6.25 | + | 6.77E-05 | 4.67E-02 |
| [pyrimidine nucleoside catabolic process](http://amigo.geneontology.org/amigo/term/GO:0046135) | [23](http://pantherdb.org/tools/gxIdsList.do?acc=GO:0046135&reflist=1) | [9](http://pantherdb.org/tools/gxIdsList.do?acc=GO:0046135&list=Client%20Text%20Box%20Input&organism=Homo%20sapiens) | 1.44 | 6.25 | + | 6.77E-05 | 4.88E-02 |
| [cytidine deamination](http://amigo.geneontology.org/amigo/term/GO:0009972) | [12](http://pantherdb.org/tools/gxIdsList.do?acc=GO:0009972&reflist=1) | [7](http://pantherdb.org/tools/gxIdsList.do?acc=GO:0009972&list=Client%20Text%20Box%20Input&organism=Homo%20sapiens) | .75 | 9.32 | + | 6.51E-05 | 4.92E-02 |
| [cytidine catabolic process](http://amigo.geneontology.org/amigo/term/GO:0006216) | [12](http://pantherdb.org/tools/gxIdsList.do?acc=GO:0006216&reflist=1) | [7](http://pantherdb.org/tools/gxIdsList.do?acc=GO:0006216&list=Client%20Text%20Box%20Input&organism=Homo%20sapiens) | .75 | 9.32 | + | 6.51E-05 | 5.16E-02 |
| [cytidine metabolic process](http://amigo.geneontology.org/amigo/term/GO:0046087) | [12](http://pantherdb.org/tools/gxIdsList.do?acc=GO:0046087&reflist=1) | [7](http://pantherdb.org/tools/gxIdsList.do?acc=GO:0046087&list=Client%20Text%20Box%20Input&organism=Homo%20sapiens) | .75 | 9.32 | + | 6.51E-05 | 5.44E-02 |
|  |  |  |  |  |  |  |  |
| [**GO molecular function complete**](http://pantherdb.org/tools/compareToRefList.jsp?sortOrder=1&sortList=categories) | [#](http://pantherdb.org/tools/compareToRefList.jsp?sortOrder=2&sortList=Homo%20sapiens) | [#](http://pantherdb.org/tools/compareToRefList.jsp?sortOrder=2&sortList=Client%20Text%20Box%20Input&sortField=num) | [expected](http://pantherdb.org/tools/compareToRefList.jsp?sortOrder=2&sortList=Client%20Text%20Box%20Input&sortField=exp) | [Fold Enrichment](http://pantherdb.org/tools/compareToRefList.jsp?sortOrder=2&sortList=Client%20Text%20Box%20Input&sortField=foldEnrich) | [+/-](http://pantherdb.org/tools/compareToRefList.jsp?sortOrder=1&sortList=Client%20Text%20Box%20Input&sortField=rep) | [raw P value](http://pantherdb.org/tools/compareToRefList.jsp?sortOrder=1&sortList=Client%20Text%20Box%20Input&sortField=pval) | 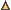 [FDR](http://pantherdb.org/tools/compareToRefList.jsp?sortOrder=2&sortList=Client%20Text%20Box%20Input&sortField=fdr) |
| [calcium ion binding](http://amigo.geneontology.org/amigo/term/GO:0005509) | [733](http://pantherdb.org/tools/gxIdsList.do?acc=GO:0005509&reflist=1) | [95](http://pantherdb.org/tools/gxIdsList.do?acc=GO:0005509&list=Client%20Text%20Box%20Input&organism=Homo%20sapiens) | 45.88 | 2.07 | + | 3.81E-10 | 1.82E-06 |
| [olfactory receptor activity](http://amigo.geneontology.org/amigo/term/GO:0004984) | [440](http://pantherdb.org/tools/gxIdsList.do?acc=GO:0004984&reflist=1) | [3](http://pantherdb.org/tools/gxIdsList.do?acc=GO:0004984&list=Client%20Text%20Box%20Input&organism=Homo%20sapiens) | 27.54 | .11 | - | 1.62E-08 | 3.87E-05 |
| [binding](http://amigo.geneontology.org/amigo/term/GO:0005488) | [16634](http://pantherdb.org/tools/gxIdsList.do?acc=GO:0005488&reflist=1) | [1110](http://pantherdb.org/tools/gxIdsList.do?acc=GO:0005488&list=Client%20Text%20Box%20Input&organism=Homo%20sapiens) | 1041.07 | 1.07 | + | 1.94E-06 | 2.32E-03 |
| [G protein-coupled receptor activity](http://amigo.geneontology.org/amigo/term/GO:0004930) | [884](http://pantherdb.org/tools/gxIdsList.do?acc=GO:0004930&reflist=1) | [23](http://pantherdb.org/tools/gxIdsList.do?acc=GO:0004930&list=Client%20Text%20Box%20Input&organism=Homo%20sapiens) | 55.33 | .42 | - | 1.68E-06 | 2.68E-03 |
| Unclassified | [2468](http://pantherdb.org/tools/gxIdsList.do?acc=UNCLASSIFIED&reflist=1) | [102](http://pantherdb.org/tools/gxIdsList.do?acc=UNCLASSIFIED&list=Client%20Text%20Box%20Input&organism=Homo%20sapiens) | 154.46 | .66 | - | 5.17E-06 | 4.12E-03 |
| [molecular_function](http://amigo.geneontology.org/amigo/term/GO:0003674) | [18383](http://pantherdb.org/tools/gxIdsList.do?acc=GO:0003674&reflist=1) | [1203](http://pantherdb.org/tools/gxIdsList.do?acc=GO:0003674&list=Client%20Text%20Box%20Input&organism=Homo%20sapiens) | 1150.54 | 1.05 | + | 5.17E-06 | 4.94E-03 |
| [deoxycytidine deaminase activity](http://amigo.geneontology.org/amigo/term/GO:0047844) | [8](http://pantherdb.org/tools/gxIdsList.do?acc=GO:0047844&reflist=1) | [7](http://pantherdb.org/tools/gxIdsList.do?acc=GO:0047844&list=Client%20Text%20Box%20Input&organism=Homo%20sapiens) | .50 | 13.98 | + | 1.02E-05 | 6.99E-03 |
| [CXCR chemokine receptor binding](http://amigo.geneontology.org/amigo/term/GO:0045236) | [18](http://pantherdb.org/tools/gxIdsList.do?acc=GO:0045236&reflist=1) | [9](http://pantherdb.org/tools/gxIdsList.do?acc=GO:0045236&list=Client%20Text%20Box%20Input&organism=Homo%20sapiens) | 1.13 | 7.99 | + | 1.48E-05 | 7.85E-03 |
| [transferase activity, transferring sulfur-containing groups](http://amigo.geneontology.org/amigo/term/GO:0016782) | [73](http://pantherdb.org/tools/gxIdsList.do?acc=GO:0016782&reflist=1) | [17](http://pantherdb.org/tools/gxIdsList.do?acc=GO:0016782&list=Client%20Text%20Box%20Input&organism=Homo%20sapiens) | 4.57 | 3.72 | + | 1.73E-05 | 8.29E-03 |
| [GABA-gated chloride ion channel activity](http://amigo.geneontology.org/amigo/term/GO:0022851) | [13](http://pantherdb.org/tools/gxIdsList.do?acc=GO:0022851&reflist=1) | [8](http://pantherdb.org/tools/gxIdsList.do?acc=GO:0022851&list=Client%20Text%20Box%20Input&organism=Homo%20sapiens) | .81 | 9.83 | + | 1.45E-05 | 8.64E-03 |
| [ligand-gated anion channel activity](http://amigo.geneontology.org/amigo/term/GO:0099095) | [19](http://pantherdb.org/tools/gxIdsList.do?acc=GO:0099095&reflist=1) | [9](http://pantherdb.org/tools/gxIdsList.do?acc=GO:0099095&list=Client%20Text%20Box%20Input&organism=Homo%20sapiens) | 1.19 | 7.57 | + | 2.06E-05 | 8.97E-03 |
| [inhibitory extracellular ligand-gated ion channel activity](http://amigo.geneontology.org/amigo/term/GO:0005237) | [15](http://pantherdb.org/tools/gxIdsList.do?acc=GO:0005237&reflist=1) | [8](http://pantherdb.org/tools/gxIdsList.do?acc=GO:0005237&list=Client%20Text%20Box%20Input&organism=Homo%20sapiens) | .94 | 8.52 | + | 3.13E-05 | 1.25E-02 |
| [glucuronosyltransferase activity](http://amigo.geneontology.org/amigo/term/GO:0015020) | [33](http://pantherdb.org/tools/gxIdsList.do?acc=GO:0015020&reflist=1) | [11](http://pantherdb.org/tools/gxIdsList.do?acc=GO:0015020&list=Client%20Text%20Box%20Input&organism=Homo%20sapiens) | 2.07 | 5.33 | + | 3.59E-05 | 1.32E-02 |
| [benzodiazepine receptor activity](http://amigo.geneontology.org/amigo/term/GO:0008503) | [11](http://pantherdb.org/tools/gxIdsList.do?acc=GO:0008503&reflist=1) | [7](http://pantherdb.org/tools/gxIdsList.do?acc=GO:0008503&list=Client%20Text%20Box%20Input&organism=Homo%20sapiens) | .69 | 10.17 | + | 4.33E-05 | 1.48E-02 |
| [cytidine deaminase activity](http://amigo.geneontology.org/amigo/term/GO:0004126) | [12](http://pantherdb.org/tools/gxIdsList.do?acc=GO:0004126&reflist=1) | [7](http://pantherdb.org/tools/gxIdsList.do?acc=GO:0004126&list=Client%20Text%20Box%20Input&organism=Homo%20sapiens) | .75 | 9.32 | + | 6.51E-05 | 2.07E-02 |
| [GABA-A receptor activity](http://amigo.geneontology.org/amigo/term/GO:0004890) | [19](http://pantherdb.org/tools/gxIdsList.do?acc=GO:0004890&reflist=1) | [8](http://pantherdb.org/tools/gxIdsList.do?acc=GO:0004890&list=Client%20Text%20Box%20Input&organism=Homo%20sapiens) | 1.19 | 6.73 | + | 1.15E-04 | 3.43E-02 |
| [metal ion binding](http://amigo.geneontology.org/amigo/term/GO:0046872) | [4289](http://pantherdb.org/tools/gxIdsList.do?acc=GO:0046872&reflist=1) | [327](http://pantherdb.org/tools/gxIdsList.do?acc=GO:0046872&list=Client%20Text%20Box%20Input&organism=Homo%20sapiens) | 268.44 | 1.22 | + | 1.46E-04 | 3.88E-02 |
| [deaminase activity](http://amigo.geneontology.org/amigo/term/GO:0019239) | [33](http://pantherdb.org/tools/gxIdsList.do?acc=GO:0019239&reflist=1) | [10](http://pantherdb.org/tools/gxIdsList.do?acc=GO:0019239&list=Client%20Text%20Box%20Input&organism=Homo%20sapiens) | 2.07 | 4.84 | + | 1.56E-04 | 3.92E-02 |
| [ion binding](http://amigo.geneontology.org/amigo/term/GO:0043167) | [6397](http://pantherdb.org/tools/gxIdsList.do?acc=GO:0043167&reflist=1) | [466](http://pantherdb.org/tools/gxIdsList.do?acc=GO:0043167&list=Client%20Text%20Box%20Input&organism=Homo%20sapiens) | 400.37 | 1.16 | + | 1.65E-04 | 3.95E-02 |
| [cation binding](http://amigo.geneontology.org/amigo/term/GO:0043169) | [4376](http://pantherdb.org/tools/gxIdsList.do?acc=GO:0043169&reflist=1) | [333](http://pantherdb.org/tools/gxIdsList.do?acc=GO:0043169&list=Client%20Text%20Box%20Input&organism=Homo%20sapiens) | 273.88 | 1.22 | + | 1.42E-04 | 3.99E-02 |
| [aryl sulfotransferase activity](http://amigo.geneontology.org/amigo/term/GO:0004062) | [10](http://pantherdb.org/tools/gxIdsList.do?acc=GO:0004062&reflist=1) | [6](http://pantherdb.org/tools/gxIdsList.do?acc=GO:0004062&list=Client%20Text%20Box%20Input&organism=Homo%20sapiens) | .63 | 9.59 | + | 1.98E-04 | 4.51E-02 |
|  |  |  |  |  |  |  |  |
| [**GO cellular component complete**](http://pantherdb.org/tools/compareToRefList.jsp?sortOrder=1&sortList=categories) | [#](http://pantherdb.org/tools/compareToRefList.jsp?sortOrder=2&sortList=Homo%20sapiens) | [#](http://pantherdb.org/tools/compareToRefList.jsp?sortOrder=2&sortList=Client%20Text%20Box%20Input&sortField=num) | [expected](http://pantherdb.org/tools/compareToRefList.jsp?sortOrder=2&sortList=Client%20Text%20Box%20Input&sortField=exp) | [Fold Enrichment](http://pantherdb.org/tools/compareToRefList.jsp?sortOrder=2&sortList=Client%20Text%20Box%20Input&sortField=foldEnrich) | [+/-](http://pantherdb.org/tools/compareToRefList.jsp?sortOrder=1&sortList=Client%20Text%20Box%20Input&sortField=rep) | [raw P value](http://pantherdb.org/tools/compareToRefList.jsp?sortOrder=1&sortList=Client%20Text%20Box%20Input&sortField=pval) | [FDR](http://pantherdb.org/tools/compareToRefList.jsp?sortOrder=2&sortList=Client%20Text%20Box%20Input&sortField=fdr) |
| [integral component of plasma membrane](http://amigo.geneontology.org/amigo/term/GO:0005887) | [1656](http://pantherdb.org/tools/gxIdsList.do?acc=GO:0005887&reflist=1) | [149](http://pantherdb.org/tools/gxIdsList.do?acc=GO:0005887&list=Client%20Text%20Box%20Input&organism=Homo%20sapiens) | 103.64 | 1.44 | + | 2.24E-05 | 4.50E-02 |
| 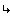[intrinsic component of plasma membrane](http://amigo.geneontology.org/amigo/term/GO:0031226) | [1734](http://pantherdb.org/tools/gxIdsList.do?acc=GO:0031226&reflist=1) | [154](http://pantherdb.org/tools/gxIdsList.do?acc=GO:0031226&list=Client%20Text%20Box%20Input&organism=Homo%20sapiens) | 108.53 | 1.42 | + | 2.68E-05 | 2.69E-02 |
|  |  |  |  |  |  |  |  |
| [**PANTHER Protein Class**](http://pantherdb.org/tools/compareToRefList.jsp?sortOrder=1&sortList=categories) | [#](http://pantherdb.org/tools/compareToRefList.jsp?sortOrder=2&sortList=Homo%20sapiens) | [#](http://pantherdb.org/tools/compareToRefList.jsp?sortOrder=2&sortList=Client%20Text%20Box%20Input&sortField=num) | [expected](http://pantherdb.org/tools/compareToRefList.jsp?sortOrder=2&sortList=Client%20Text%20Box%20Input&sortField=exp) | [Fold Enrichment](http://pantherdb.org/tools/compareToRefList.jsp?sortOrder=2&sortList=Client%20Text%20Box%20Input&sortField=foldEnrich) | [+/-](http://pantherdb.org/tools/compareToRefList.jsp?sortOrder=1&sortList=Client%20Text%20Box%20Input&sortField=rep) | [raw P value](http://pantherdb.org/tools/compareToRefList.jsp?sortOrder=1&sortList=Client%20Text%20Box%20Input&sortField=pval) | 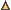 [FDR](http://pantherdb.org/tools/compareToRefList.jsp?sortOrder=2&sortList=Client%20Text%20Box%20Input&sortField=fdr) |
| [chemokine](http://pantherdb.org/panther/category.do?categoryAcc=PC00074) | [17](http://pantherdb.org/tools/gxIdsList.do?acc=PC00074&reflist=1) | [10](http://pantherdb.org/tools/gxIdsList.do?acc=PC00074&list=Client%20Text%20Box%20Input&organism=Homo%20sapiens) | 1.06 | 9.40 | + | 1.63E-06 | 3.17E-04 |
| [defense/immunity protein](http://pantherdb.org/panther/category.do?categoryAcc=PC00090) | [398](http://pantherdb.org/tools/gxIdsList.do?acc=PC00090&reflist=1) | [7](http://pantherdb.org/tools/gxIdsList.do?acc=PC00090&list=Client%20Text%20Box%20Input&organism=Homo%20sapiens) | 24.91 | .28 | - | 6.55E-05 | 6.38E-03 |
| [immunoglobulin receptor superfamily](http://pantherdb.org/panther/category.do?categoryAcc=PC00124) | [150](http://pantherdb.org/tools/gxIdsList.do?acc=PC00124&reflist=1) | [0](http://pantherdb.org/tools/gxIdsList.do?acc=PC00124&list=Client%20Text%20Box%20Input&organism=Homo%20sapiens) | 9.39 | < 0.01 | - | 1.77E-04 | 1.15E-02 |
| [cytokine](http://pantherdb.org/panther/category.do?categoryAcc=PC00083) | [81](http://pantherdb.org/tools/gxIdsList.do?acc=PC00083&reflist=1) | [15](http://pantherdb.org/tools/gxIdsList.do?acc=PC00083&list=Client%20Text%20Box%20Input&organism=Homo%20sapiens) | 5.07 | 2.96 | + | 4.76E-04 | 2.32E-02 |
|  |  |  |  |  |  |  |  |
| [**PANTHER Pathways**](http://pantherdb.org/tools/compareToRefList.jsp?sortOrder=1&sortList=categories) | [#](http://pantherdb.org/tools/compareToRefList.jsp?sortOrder=2&sortList=Homo%20sapiens) | [#](http://pantherdb.org/tools/compareToRefList.jsp?sortOrder=2&sortList=Client%20Text%20Box%20Input&sortField=num) | [expected](http://pantherdb.org/tools/compareToRefList.jsp?sortOrder=2&sortList=Client%20Text%20Box%20Input&sortField=exp) | [Fold Enrichment](http://pantherdb.org/tools/compareToRefList.jsp?sortOrder=2&sortList=Client%20Text%20Box%20Input&sortField=foldEnrich) | [+/-](http://pantherdb.org/tools/compareToRefList.jsp?sortOrder=1&sortList=Client%20Text%20Box%20Input&sortField=rep) | [raw P value](http://pantherdb.org/tools/compareToRefList.jsp?sortOrder=1&sortList=Client%20Text%20Box%20Input&sortField=pval) | 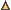 [FDR](http://pantherdb.org/tools/compareToRefList.jsp?sortOrder=2&sortList=Client%20Text%20Box%20Input&sortField=fdr) |
| [Cadherin signaling pathway](javascript:openDiagramWindow('/pathway/pathwayDiagram.jsp?color=1&catsInfo=true&catAccession=P00012');) | [160](http://pantherdb.org/tools/gxIdsList.do?acc=P00012&reflist=1) | [58](http://pantherdb.org/tools/gxIdsList.do?acc=P00012&list=Client%20Text%20Box%20Input&organism=Homo%20sapiens) | 10.01 | 5.79 | + | 6.28E-23 | 1.03E-20 |
| [Wnt signaling pathway](javascript:openDiagramWindow('/pathway/pathwayDiagram.jsp?color=1&catsInfo=true&catAccession=P00057');) | [317](http://pantherdb.org/tools/gxIdsList.do?acc=P00057&reflist=1) | [72](http://pantherdb.org/tools/gxIdsList.do?acc=P00057&list=Client%20Text%20Box%20Input&organism=Homo%20sapiens) | 19.84 | 3.63 | + | 2.74E-18 | 2.24E-16 |
| Unclassified | [18243](http://pantherdb.org/tools/gxIdsList.do?acc=UNCLASSIFIED&reflist=1) | [1072](http://pantherdb.org/tools/gxIdsList.do?acc=UNCLASSIFIED&list=Client%20Text%20Box%20Input&organism=Homo%20sapiens) | 1141.77 | .94 | - | 8.65E-08 | 4.73E-06 |
